# Frequency Response of a Protein to Local Conformational Perturbations

**DOI:** 10.1371/journal.pcbi.1003238

**Published:** 2013-09-26

**Authors:** Dilek Eren, Burak Alakent

**Affiliations:** Department of Chemical Engineering, Bogazici University, Bebek, Istanbul, Turkey; University of Wisconsin-Madison, United States of America

## Abstract

Signals created by local perturbations are known to propagate long distances through proteins via backbone connectivity and nonbonded interactions. In the current study, signal propagation from the flexible ligand binding loop to the rest of Protein Tyrosine Phosphatase 1B (PTP1B) was investigated using frequency response techniques. Using restrained Targeted Molecular Dynamics (TMD) potential on WPD and R loops, PTP1B was driven between its crystal structure conformations at different frequencies. Propagation of the local perturbation signal was manifested via peaks at the fundamental frequency and upper harmonics of 1/*f* distributed spectral density of atomic variables, such as C_α_ atoms, dihedral angles, or polar interaction distances. Frequency of perturbation was adjusted high enough (simulation length >∼10×period of a perturbation cycle) not to be clouded by random diffusional fluctuations, and low enough (<∼0.8 ns^−1^) not to attenuate the propagating signal and enhance the contribution of the side-chains to the dissipation of the signals. Employing Discrete Fourier Transform (DFT) to TMD simulation trajectories of 16 cycles of conformational transitions at periods of 1.2 to 5 ns yielded C_α_ displacements consistent with those obtained from crystal structures. Identification of the perturbed atomic variables by statistical t-tests on log-log scale spectral densities revealed the extent of signal propagation in PTP1B, while phase angles of the filtered trajectories at the fundamental frequency were used to cluster collectively fluctuating elements. Hydrophobic interactions were found to have a higher contribution to signal transduction between side-chains compared to the role of polar interactions. Most of in-phase fluctuating residues on the signaling pathway were found to have high identity among PTP domains, and located over a wide region of PTP1B including the allosteric site. Due to its simplicity and efficiency, the suggested technique may find wide applications in identification of signaling pathways of different proteins.

## Author Summary

Similar to a machine in which interactions between different parts determine its function, signaling between the residues of a protein may play an important role in determining its function. External perturbations, such as ligand binding to a local region, may trigger a global response of the protein, manifested as perturbations in positions or mobility of atoms. Here we introduce a frequency response technique, in which a local periodic perturbation is employed on a flexible loop of a protein, and atomic responses are analyzed. Protein response characteristics are found to be closely related to perturbation frequency, so frequency analysis tools such as power spectral densities and magnitude Bode plots are utilized. Conformational change of the protein estimated by this method is found to be consistent with that determined from crystal structures. We cluster the phase angles of side-chains dihedral angles to identify collectively fluctuating residues, and determine a large number of hydrophobic interactions, which help intraprotein signal propagation. We believe that the suggested frequency response technique will be a fine contribution to the existing repertoire of perturbation methods.

## Introduction

Proteins are molecular machines with a variety of functions facilitated by their intrinsic flexibility and dynamics [1]–[4]. Driven by nonlinear atomic interactions, protein dynamics span a wide range in time and spatial scale; hence protein fluctuations may be viewed as nonperiodic transitions between hierarchically organized conformational substates [5]–[7]. Protein conformations and dynamics are sensitive to various external disturbances, such as changes in environment temperature [8], [9], ligand binding [10], [11], or post-translational modifications [12]. According to population shift paradigm, changing the surrounding environment, e.g. via ligand binding, shifts the energy landscape of a protein, redistributing the already existing populations of substates [13]. In expansion of this view, it was suggested that external disturbances may also be transmitted as changes in dynamic fluctuations without significant variation in backbone conformation [14].

Signal produced by local interaction with a ligand may reach distant sites of the protein and this propagation mechanism of regulatory signals is known as allostery [15]. Long range communications in proteins have often been interpreted referring to global (or collective) dynamics. A general method of elucidating global motions comprises performing equilibrium Molecular Dynamics (EMD) simulations on various states of proteins, such as ligation states, and applying linear statistical methods, such as correlation analysis and principal component analysis (PCA), on the resulting atomic trajectories [16]–[18]. Recently, novel statistical methods have been employed to elucidate the roles of nonlinear and non-Gaussian components in proteins dynamics [4]. For instance, nonlinear PCA was applied on peptides to increase the percentage of explained fluctuations in the low-dimensional space [19]; isomap algorithm was employed on folding simulation of coarse-grained model of SH3 domain to represent the intrinsic dynamics on a nonlinear manifold [20], and independent component analysis was applied on T4 lysozyme to determine collective motions with non-Gaussian distributions [21], [22]. Another important aspect of global dynamics is vibrational frequencies of collective modes, and various approaches, such as Normal Mode Analysis (NMA) [23] and time series models [24], have been used to investigate how distribution of vibrational frequencies may change upon ligand binding.

Linear and nonlinear feature extraction methods yield collective dynamics at global scale, thus obtained results are often at low resolution. Elucidation of functionally important motions in proteins, however, demands more detailed analyses which would determine the dynamical roles of individual structural elements and residues. Network analyses showed the significance of protein topology on information flow [25], so topology information has been incorporated into residue communication analyses. Interaction-correlation matrices of active and inactive states of rhodopsin were determined using nonbonded energy fluctuations of residue pairs during equilibrium MD simulations, and signaling pathways were found to be different in two states of the protein [26]. Application of local feature analysis (LFA), which extracts sparsely distributed collective motions, on T4 lysozyme gave a clearer picture of how different parts of the protein may be moving compared to that obtained by the sole application of PCA [27]. Recently, LFA and variance of inter-residue distances during the simulations were utilized to determine independent dynamic segments and allosteric communications in KIT receptor tyrosine kinase [28]. In another interesting study, direction of information flow between residues was determined using information theory techniques [29].

Despite the progress in processor power in the recent years, equilibrium MD simulations still cannot be extended to time scales of functionally important conformational motions, and this prevents identification of rarely-occurring and/or subtle functionally important atomic motions. MD perturbation methods are used to overcome the difficulties encountered by equilibrium simulations in elucidating how information is propagated between distant sites and identify key residues contributing to these communication paths. Anisotropic Thermal Diffusion (ATD) [30] and Pump-Probe Molecular Dynamics (PPMD) methods [31] are two of the pioneering examples of MD perturbation methods, in which external perturbations were applied on local regions of proteins to elucidate intramolecular signaling. In the former study, a PDZ domain protein was equilibrated to 10 K while the target part was coupled to a heat bath at 300 K, and propagation of energy throughout the protein, quantified by root mean square deviation (RMSD) from the minimized structure, was monitored. In the latter study, an oscillating force of specified magnitude was applied on C_α_ atoms of a PDZ domain protein to induce a circular motion around an arbitrary axis, and couplings between C_α_ atoms were determined. These studies aimed to explain the mechanism of anisotropic energy transport in proteins by suggesting energy transport channels comprising residues [32]. While both studies showed long range couplings between residues, most of the perturbation energy was transferred through the backbone, and side-chains were not perturbed sufficiently to determine their contribution to intraprotein signaling [33]. In Rotamerically Induced Perturbation (RIP) method developed to remedy this problem [34], periodic perturbations at amplitudes of 60° were applied to side-chains of all residues and significant residues in communication pathways were identified. In another study focusing on side-chains, Monte Carlo samplings of side-chain dihedral angles were performed on proteins with fixed backbones, and single side-chain perturbations were found to be transmitted to long distances [35]. Instead of atomistic MD simulations, simplified networks of C_α_ atoms are used in Perturbation-Response Scanning (PRS) methods, and residues with significant contribution to displacements were determined by applying random forces to different nodes [36], [37]. Dynamic character is given to network perturbation methods using Markovian transmission models, in which relaxation of residues upon a disturbance in initial conditions was monitored [38].

While having substantially increased our understanding of intraprotein signaling, it is difficult to achieve unbiased contribution of backbone and side-chains to signal transduction using existing MD perturbation methods [30], [31], [34], [35], and parameters, such as frequency, directionality and magnitude of applied perturbations, have not been thoroughly elucidated. In order to offer plausible solutions to these problems, we employed a novel frequency response technique on Protein Tyrosine Phosphate 1B (PTP1B) in the current study. PTP1B is a member of Protein Tyrosine Phosphatase (PTP) family, which removes the phosphate group from phosphotyrosine (pTyr) residues [39], [40], and an important target for diabetes, obesity and cancer [41]–[43]. While the majority of crystal structures in Protein Data Bank (PDB) showed that its flexible WPD loop adopted closed active (WDP_closed_) and open inactive (WDP_open_) conformations in the ligand bound and free states of PTP1B (Figure S1), respectively [44], [45], there exist a number of liganded WPD_open_ and free WPD_closed_ structures [44], [46]. Furthermore, WPD loop of *Yersinia* PTP was shown to adopt open and closed conformations in both free and liganded states in submilisecond scale [47]. Inhibition of PTP1B via an inhibitor bound to a site, which is ∼20 Å distant to the active site, established allosteric inhibition in PTP1B [48]. Truncation of α7 on the C-terminus of the resolved PTP1B was found to decrease the activity of PTP1B, while mutations on α7 were found to reduce the potency of inhibitors [49]. Adding C-terminal domain of PTP1B to N-terminal domain was found to influence the activity of the enzyme [50]. Computational studies also revealed fine details of the collective motions in PTP1B, such as coupling of WPD loop with α3, α6, L11 and active site waters [51]–[53].

In this study, we employed restrained Targeted MD (TMD) potential on WPD and R loops of PTP1B, and altered the target function between WDP_open_ and WDP_closed_ conformations at different frequencies. Discrete Fourier Transform (DTF) was applied on the raw trajectories of atomic variables, such as C_α_ atomic displacements, dihedral angles of side-chains and backbone, and distance of polar interactions. Perturbed atomic variables were determined using statistical t-test, which detected deviations from the linear slope of the log-log scale spectral densities. Reconstructed trajectories of atomic variables at the fundamental frequency were used to predict the conformational response of the protein to the local disturbance, and phase angles of the reconstructed trajectories were utilized to determine dynamically coupled residues, which may play roles in signal propagation from the flexible ligand binding loop of PTP1B. Four clusters of in-phase fluctuating residues with inter-cluster phase differences of π/4 were identified. The first cluster of residues made coupled fluctuations with the WPD loop, while most of the residues in the second and third cluster moved collectively with the R-loop. Phase difference between WDP and R loops was found to be consistent with the experimental structures and the suggested mechanism of WPD loop closure in the literature. Hydrophobic interacting side-chains, backbone connectivity, intra- and inter-backbone H-bonds were found to be the major actors in signal transduction within clusters, with minor contributions from side-chain polar interactions.

## Results

Results are organized as follows. In the first section, method of trajectory reconstruction to study the effects of perturbation signal is explained. Experimental verification of the suggested method is presented in the following section. In the third and fourth sections, perturbed atomic variables are identified, and then clustered with respect to their phase angles. Effect of perturbation frequency on fluctuation amplitudes is elucidated in the final section.

### Separating local disturbance signal from random atomic fluctuations

**Figure 1:**
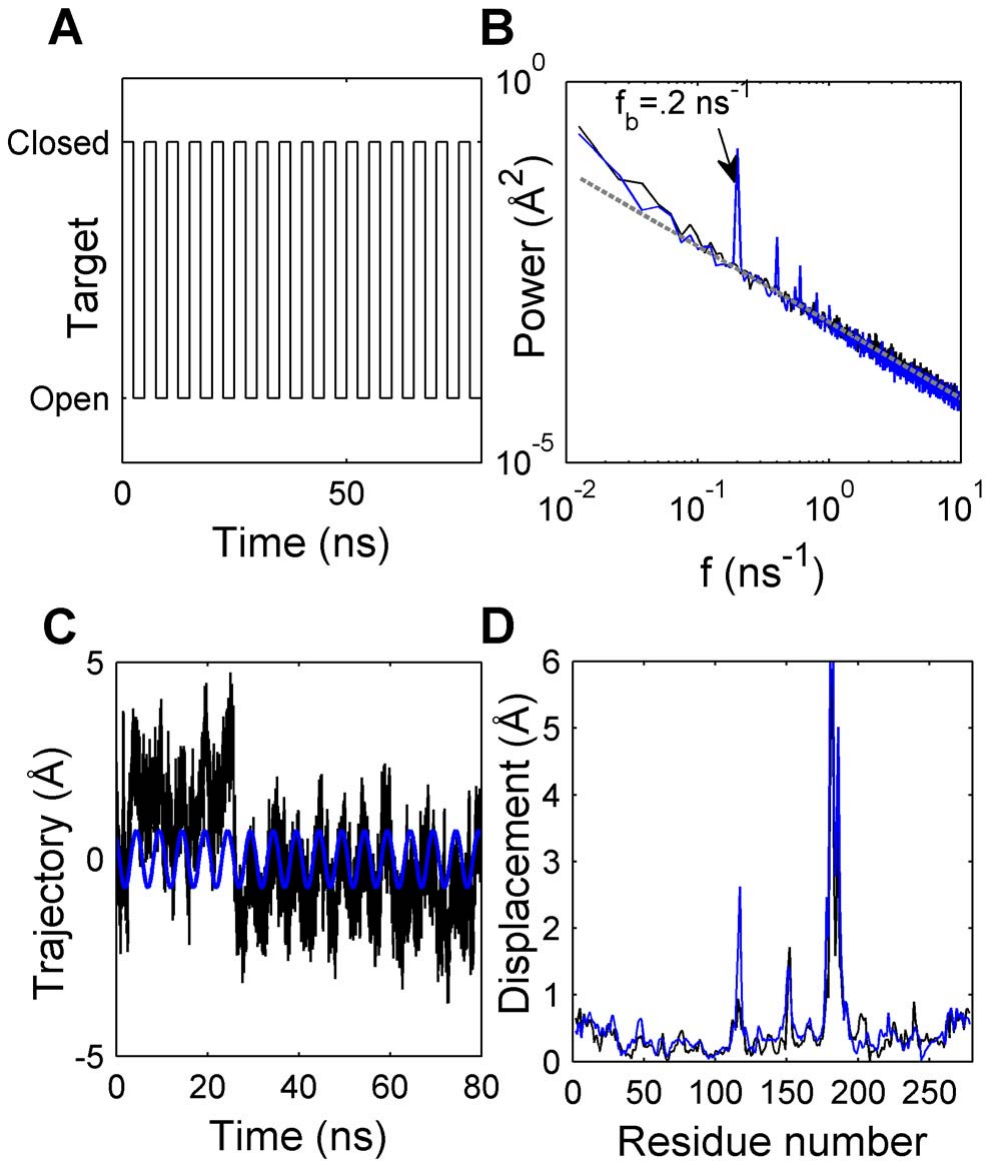
Frequency components of C_α_ trajectories in EMD and TMD_1_ simulations. (A) Time evolution of the target function in TMD_1_ simulation. (B) Power spectral density per residue (or residue-averaged MSF) for residues 2 to 278. Frequency components of EMD and TMD_1_ simulations are represented with black and blue solid lines, respectively. Gray dashed lines represent the least-squares lines fit to power spectrum. (C) Reconstructed trajectory of z-Cartesian coordinate of Ser151 C_α_ atom using power at the base frequency in TMD_1_ simulation. Raw and reconstructed trajectories are shown in black and blue, respectively. (D) Amplitudes of C_α_ displacements determined from WPD_open_ and WPD_closed_ crystal structures (black) and those estimated using reconstructed in-phase trajectories (blue).

TMD_1_ and EMD (see Materials and Methods) simulations both were sampled at 1 ps for simulation periods of 80 ns. The target structure in TMD_1_ simulation was altered between WPD_open_ and WPD_closed_ structures at 2.5 ns intervals, thus the frequency of the targeting function (*f_0_*) was 0.2 ns^−1^ (Figure 1A). The highly mobile region comprising residues 281 to 298 (α7 and the loop connecting α6 and α7) was not included in the structural analyses because of the disordered nature of this region [44], [52]. Aligning the C_α_ atoms of Glu2 to Ala278, RMSD from the crystal structure of PTP1B leveled off between 1.5 and 2.0 Å, showing that protein structure was maintained in both simulations (Figure S2A). In EMD simulation, WPD loop did not approach to its WPD_closed_ structure (Figure S2B), while periodicity of the WPD loop opening/closing motions was clearly observed in TMD_1_ (Figure S2C). A line of slope −1.0 fits perfectly to all but the first ∼10–12 frequency components in the range of log-log plot of residue-averaged power spectral density [54] in EMD and TMD_1_ simulations (Figure 1B). Power (*P*) spectra of both simulations overlap well, particularly for *f*>0.15 ns^−1^ (12^th^ Fourier component). The spectrum of TMD_1_ shows peaks at the fundamental (base) frequency (*f_b_*) 0.2 ns^−1^, equal to the TMD_1_ target function frequency, and at upper harmonics (*f_u_*), at 0.4, 0.6 and 0.8 ns^−1^ (Figure S3).

Power at the fundamental frequency in TMD simulations is used to distinguish the effect of perturbation signal from thermal fluctuations. Making all the frequencies expect the fundamental frequency (and its symmetrical component) zero and applying inverse DFT for all C_α_ atomic trajectories, filtered (or reconstructed) atomic coordinates are obtained. Benefit of the filtering method may be better appreciated on trajectories of residues which do not reside on WPD loop. For instance, diffusive motion of Ser151 C_α_ atom on L11 clouded the local perturbation signal on its z-component trajectory, so periodic fluctuations can be observed only in its filtered trajectory, explaining 14% of fluctuations (Figure 1C). Statistical significance of the power observed at the base frequency in TMD simulations will be discussed thoroughly in the following sections, nonetheless it should here be emphasized that the same frequency component in EMD simulation explained only ∼0.05% of the total fluctuations. This observation strongly suggests that the perturbation signal has been transduced to L11, and though the contribution of noise to atomic fluctuations may be high, signal may be distinguished from thermal noise.

### Comparison of predicted and experimental residue displacements

A single C_α_ displacement vector representing the collective conformational change of the whole PTP1B was obtained via applying PCA on the reconstructed trajectories (Text S1 and Figure S4), yielding reconstructed in-phase trajectories of C_α_ atoms. Experimental residue displacements were obtained from an initial set of 58 crystal structures (Table S1) from Protein Data Bank (PDB), and this set was further reduced to 36 structures in order to overcome the bias introduced by L16 region, which adopts two different conformations in both WPD_open and_ WPD_closed_ crystal structures (Text S2 and Figure S5,S6). Correlation of experimental and predicted magnitude of atomic displacements (Figure 1D) was found to be 0.97 and 0.76 for all C_α_ atoms and all C_α_ atoms excluding the TMD potential applied region, respectively. Average residue displacements (excluding R and WPD loops) were found to be 0.38 Å and 0.34 Å in TMD_1_ simulation and crystal structures, respectively, showing the consistency of predictions with experimental findings.

Directions of residue displacements during the conformational transition of PTP1B determined from the reconstructed in-phase signals and crystal structures were also found to be in agreement. Overlap of the first eigenvector (*p*) of the reconstructed trajectories with the atomic displacement vector determined from crystal structures (*d*) is 0.86 and 0.66 for all C_α_ atoms and all C_α_ atoms excluding the TMD potential applied region, respectively (Table 1). To evaluate the quality of our predictions, we also performed PCA [2], [3] and linear response theory (LRT) method [55], [56] on the EMD trajectory. Overlap of single eigenvectors with the experimental displacements was found to be very low; only by taking the essential dynamics subspace composed of twenty principle components (PCs), overlap values reached 0.59. Mimicking the local interaction by an external force between C_α_ atom of Asp181 on WPD loop and the center of mass of PO_4_ in the WDP_closed_ crystal structure, overlap of LRT predictions with experimental displacements was found to be 0.62. Accuracy of predictions of the current method is easily on par with those from conventional methods, hence an initial confirmation of the frequency response method was obtained. The current method was also found to be robust to perturbation frequencies, as direction of atomic displacements was seen to be unaltered to perturbations employed at periods of 2 ns and 1.2 ns (Figure S7 and Table S2). Relation between perturbation frequency and protein response is elaborated in the last section, nonetheless it should here be emphasized that similarity of atomic responses is confined to low frequency perturbations only.

**Table 1:** Overlap of the residue displacements determined from the reconstructed in-phase trajectories at the base frequency, PCA, LRT method and crystal structures.

Overlap	Residues 2 to 278	R and WPD loops excluded
*d* (a) and *p*	0.86	0.66
*d* and *e_1_*	0.07	0.11
*d* and *e_6_* (b)	0.18	0.35
*d* and *e_10_*	0.34	0.13
*d* and *e_1-10_* (c)	0.56	0.35
*d* and *e_1-20_* (d)	0.72	0.59
*d* and *l* (e)	0.83	0.62

(a) *d* is the C_α_ displacement vector between the averages of WPD_open_ and WPD_closed_ crystal structures in L16_I_ conformation (see Text S2).

(b) Sixth and tenth (in the next row) eigenvectors are the single eigenvectors obtained from EMD simulation having the highest overlap with the experimental conformational change.

(c) Linear combination of the first 10 eigenvectors consistent with the conformational change of PTP1B.

(d) Linear combination of the first 20 eigenvectors consistent with the conformational change of PTP1B.

(e) *l* is the C_α_ displacement vector obtained by LRT method.

### Identification of perturbed atomic variables

**Figure 2:**
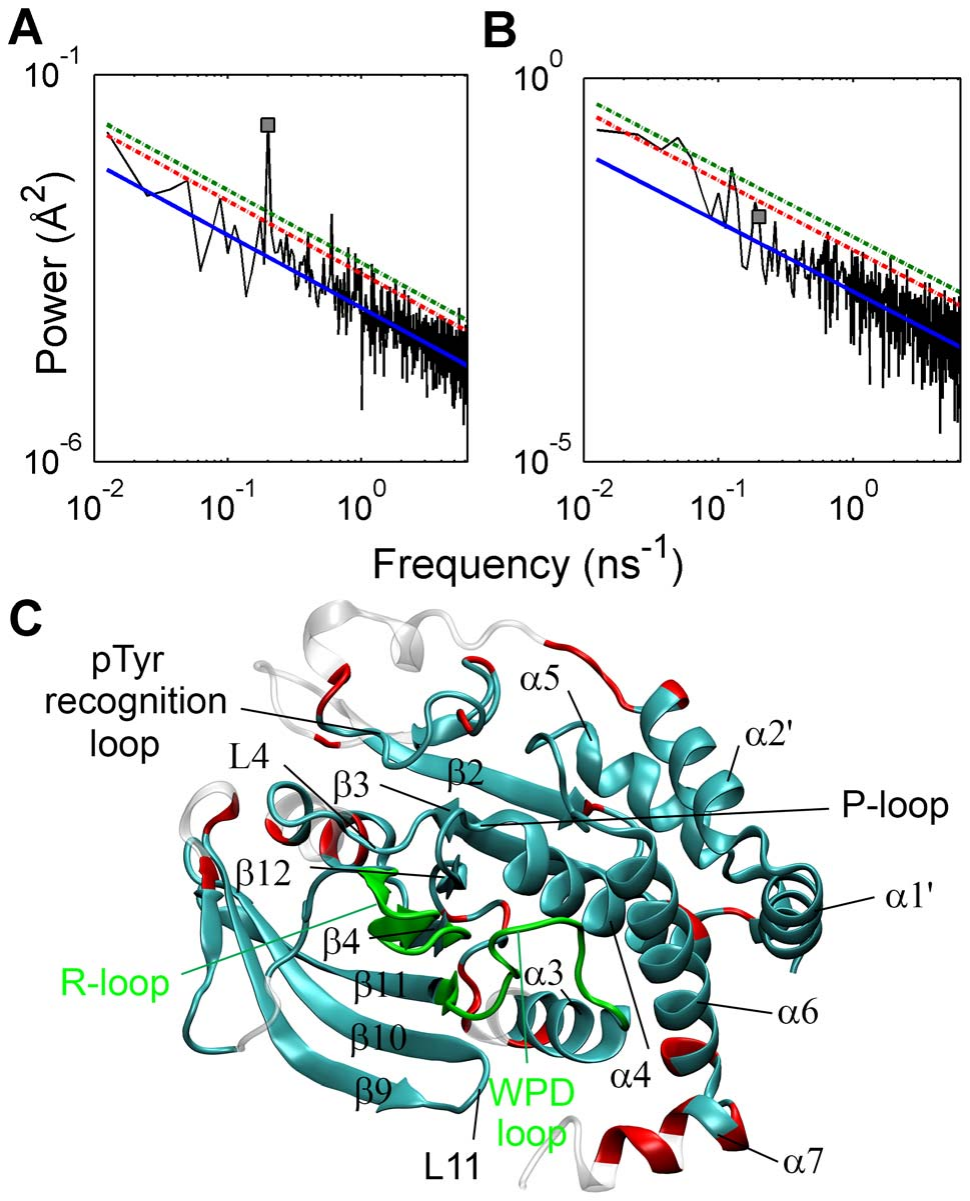
Identification of perturbed C_α_ atoms. Power spectral density of (A) Cys215 and (B) Asp63. MSF of Cys215 and Asp63 were found to be 0.17 Å^2^ and 1.97 Å^2^, respectively. In both figures, solid blue line is the least-squares line fit to the power spectrum (black), while red and green dashed lines represent the 95% and 99% confidence limit estimates, respectively, and square represents the power at the base frequency. (C) Mapping of C_α_ atoms perturbed at significance levels of 95% (red) and 99% (cyan) on the three-dimensional structure of PTP1B. Green regions are the structural elements on which TMD potential was applied.

Spectral density of C_α_ displacements (Text S3) was analyzed to detect whether power at the fundamental frequency was perturbed during WPD loop transition. To deem significance to perturbations in power spectra, a simple statistical method is suggested. Based on the observation that energy spectral density for C_α_ atomic fluctuations has ∼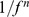 distribution expect for the lowest frequencies, a least-squares line is fit to the energy (or power) spectral density (excluding the first 10 frequency components), and one-sided upper confidence interval for energy at the base frequency is determined using the standard least-squares procedure [57]. If the power component at the base frequency exceeds this confidence interval, then the null hypothesis that energy at the base frequency has not been perturbed is rejected, showing that C_α_ fluctuations of that residue are perturbed by the local disturbance in TMD simulations. As a demonstration, power spectral densities of C_α_ atoms of Cys215 and Asp63 in TMD_1_ simulation are shown in Figure 2A,B. Cys215 is a catalytically essential residue in P-loop and ∼12 Å distant from Asp181, while Asp63 lies ∼37 Å away from Asp181. Although mobility of Asp63 was much higher than that of Cys215, power of Cys215 at the base frequency exceeded 1% confidence limit, while that of Asp63 was below the 5% limit. This indicates that the local disturbance in PTP1B increased the amplitude of Cys215 fluctuations, but did not affect Asp63. Power at the first five frequency components of Asp63 was significantly higher than the least-squares line, showing that random diffusional motion of Asp63, possibly independent of WPD loop fluctuations, may be responsible for high mobility of this residue.

Mapping the significantly perturbed residues at the base frequency to protein structure (Figure 2C) shows that a large number of residues were perturbed by the local disturbance. Fluctuation amplitudes of 87% and 77% of all residues at confidence levels of 0.95 (α = 0.05) and 0.99 (α = 0.01) [57], respectively, were found to be perturbed. Repeating the analysis at different base frequencies by changing the number of perturbation cycles (Figure S8) and length of TMD simulations (Table S3) confirmed the robustness of the identification method of perturbed residues (Text S4). Residues with unperturbed C_α_ atoms (transparent in Figure 2C) lie mainly on the opposite face of PTP1B, and on the C-termini of α3 and α7. Identification of a large number of perturbed C_α_ atoms makes it necessary to include other atomic variables in the analysis to obtain a higher resolution picture of intraprotein signaling. For this purpose, backbone dihedral angles, side-chain dihedral angles and polar interaction distances were analyzed by the same method.

**Figure 3:**
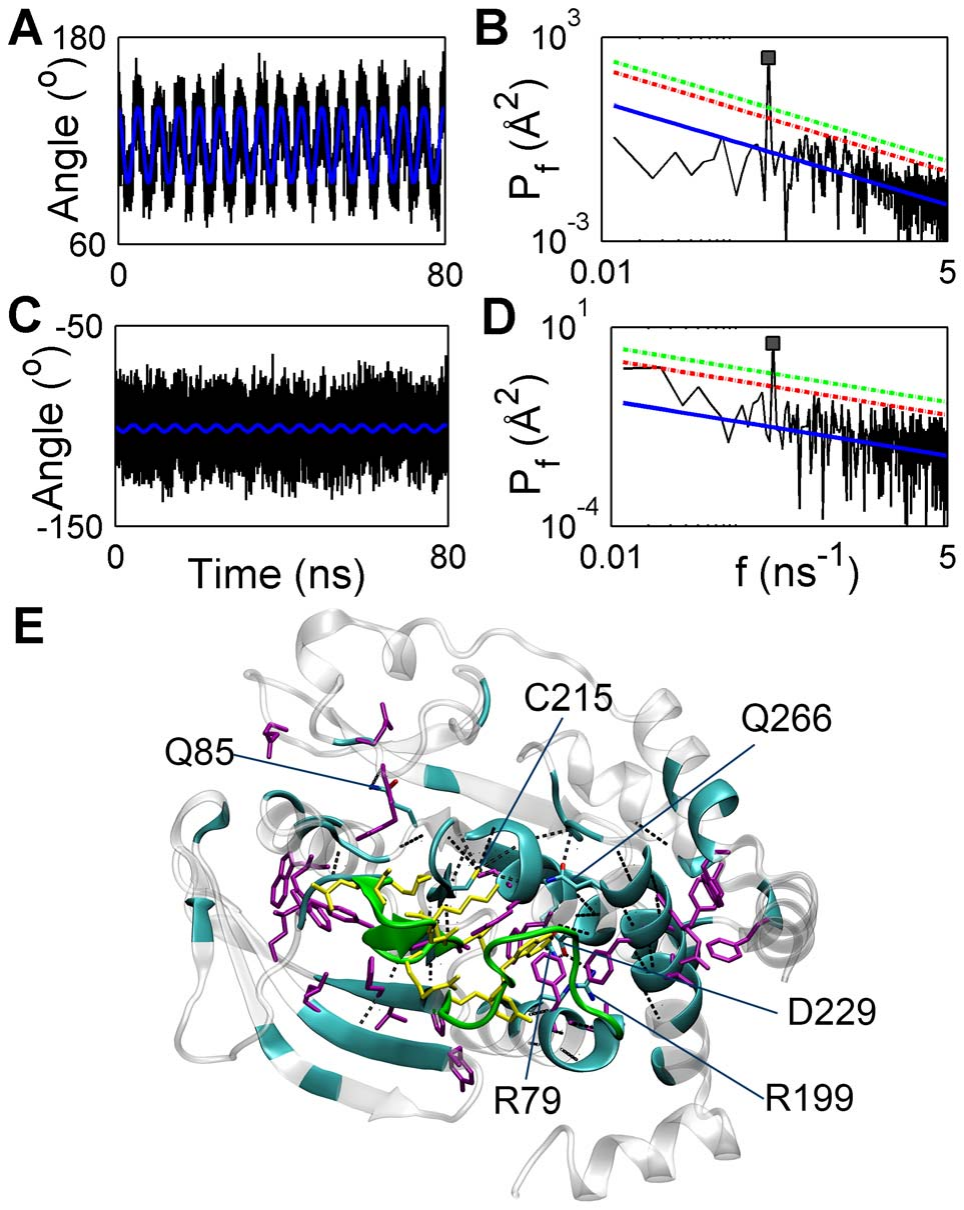
Identification of perturbed backbone and side-chain dihedral angles, and polar interactions. (A) Raw trajectory (black), reconstructed trajectory (blue), and (B) power spectral density (*P_f_*) of *ψ*_179_. (C) Raw trajectory (black), reconstructed trajectory (blue), and (D) power spectral density of *φ*_215_. Coloring and line styles in the power spectra are identical to those in Figure 2A,B. (E) Residues with perturbed backbone dihedral angles (cyan), side-chain dihedral angles (purple), H-bonds (black dashes lines) at a significance level of 95%. Residues with yellow side-chains and green backbone belong to the structural elements on which TMD potential is applied. Side-chains participating in perturbed polar interactions are colored with respect to their atom types and labeled.

Dihedral angles are represented by circular data, so each dihedral angle was transformed to a vector on a unit circle, as sine and cosine components before employing DFT on its trajectories [58]. Similar to the results obtained for atomic fluctuations, 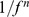 relation was captured in power spectral density of dihedral angles, as demonstrated in two examples: *ψ*_179_ at the hinge of WPD loop, and *φ*_215_ in the P-loop (Figure 3A–D). Periodic motion is already evident in the raw trajectory of *ψ*_179_, and power at 0.2 ns^−1^ captures 68% of its mean square fluctuations (MSF). Although a periodic pattern cannot be observed in the raw trajectory of *φ_215_*, a spike at 0.2 ns^−1^ in the power spectrum shows that this dihedral angle was also perturbed. In PTP1B, 33% and 21% of the backbone dihedral angles were found to be perturbed at α = 0.05 and 0.01, respectively. Excluding R and WPD loops, percent of perturbed backbone dihedral angles decreased to 25% and 13% at α = 0.05 and 0.01, respectively. Compared to the high number of perturbed C_α_ atoms, perturbed backbone dihedral angles formed a smaller cluster surrounding the active site, making it easier to analyze signal propagation (Figure 3E). Connectivity of β4, β10, β11, and β12 to the active site suggests a plausible communication path: Fluctuations in N-termini of R and WPD loops may perturb the backbone atoms on β4 and β11, which, in turn, may perturb β10 and β12 with the help of H-bonds formed between these β-strands. WPD loop conformational transition was suggested to be coupled to α3 and α6 motions in previous studies [48], [52], and N-termini of α3 and α6 were indeed found to be perturbed. Subtle but statistically significant dihedral angle changes in α-helices (∼8° in α3, and ∼4° in α6) suggest that small perturbations on the structural elements may play roles in signal transduction. This phenomenon is more vividly demonstrated in the P-loop (Table 2). Although the catalytically essential P-loop is found to be negligibly displaced between the WPD_open_ and WPD_closed_ crystal structures except in oxidized state [59], the current analysis indicates a significant increase in the mobility of the P-loop backbone dihedral angles. Considering that perturbation signal was propagated to regions surrounding the P-loop, such as L4, Q-loop and α4, subtle perturbations in the active site of PTP1B may be significant in global transduction of intraprotein signal [25].

**Table 2:** Distribution of atomic variables perturbed at α = 0.05 among structural elements of PTP1B.

Structural element	(a)C_α_ atoms	Backbone dihedral angles	Side-chain dihedral angles	Polar interactions(b)
α1′–α2′ (6–26)(c)	21	4	4	0
β4 (106–109)	4	2	2	4
β10 (154–162)	9	5	2	1
β11 (168–175)	8	5	3	4
β12 (211–214)	4	3	0	4
α3 (188–202)	12	7	3	6
α4 (222–237)	16	8	3	7
α6 (267–280)	14	9	4	5
pTyr recognition loop (44–50)	7	1	2	2
L4 (85–90)	6	2	2	4
P-loop (215–221)	7	7	2	7
Q-loop (260–266)	7	6	1	2

(a) Each column shows the number of perturbed atomic variables in each structural element except R and WPD loops.

(b) This column represents the number of residues participating in perturbed polar interactions.

(c) Numbers in parentheses show the residue numbers of each structural element.

Employing DFT on side-chain dihedral angles in PTP1B showed that power at the base frequency of 46 residues (32 of these residues reside out of R and WPD loops) was increased at α = 0.05 (Figure S9). Perturbed side-chains form a number of different clusters, such as a set of contacting residues between β10-β11-β4, or between α6-α1′-α2′ (see Figure 3E). These clusters are examined in more detail in the following section.

Finally, distances between all polar interacting atoms in PTP1B were examined by DFT. Power of 38 interatomic distances at 0.2 ns^−1^ showed significant increase at α = 0.05. 27 of these atom pairs formed H-bonds between the backbone atoms, while five of the H-bonds were formed between Cys215 and the backbone amides of P-loop, and the remaining six polar atom pairs belonged to side-chains of four different residues. H-bonds formed between the backbone atoms of β4, β10, β11, and β12 were perturbed, corroborating the previous suggestion that H-bonds may participate in the concerted motions of these four β-strands. Identification of a high number of perturbed H-bonds between backbone amide-oxygen atoms in α3, α4 and α6 indicates that H-bonds may also play a role in signal transduction along the helices (see Table 2). Perturbation of H-bonds formed between Cys215 and P-loop backbone amides is another indication of the perturbation of the P-loop. Two other perturbed H-bonds contributed by P-loop are formed with Q-loop, a catalytically important element of PTP1B [60], and L4, part of a highly conserved region among PTP family (named motif 4 [61]), indicating diffusion of perturbation signal from the active site to outer regions via H-bonds.

### Clustering of perturbed atomic variables with respect to phase angles

Fluctuation of two atomic variables at the same frequency does not guarantee a concerted (collective) motion, i.e. correlation of two C_α_ atoms fluctuating with a phase difference of π/2 at the same frequency is equal to zero. Phase angles of the reconstructed trajectories of atomic variables should be similar so that they may be presumed to fluctuate collectively. Phase angle represents the relative position of a periodic motion from an arbitrary starting point in time, and has a range of 2π. A phase angle difference of π implies two anti-correlated signals, i.e. maxima of the first signal coincide with the minima of the second signal. Residue correlation maps constructed using C_α_ displacements are based on this interpretation; correlated residues move in the same direction, while anti-correlated residues move in opposite directions [9], [16]. Unlike the Cartesian frame of reference used to compute residue displacement correlations, a dihedral angle is determined by the relative position of two planes formed by four consecutive atoms. Hence, it is not possible, without a detailed structural analysis, to suggest that two different dihedral angles with a phase difference of π are correlated or anti-correlated. Therefore in the current study, range of phase angles was limited to π and two dihedral angles fluctuating with a phase difference of zero or π were assumed to make a concerted motion. To cluster the circular phase angle data [58] in the range of π, phase angles are mapped to a two-dimensional plane of axes, cos(2*θ*) and sin(2*θ*). Hence, one rotation around the origin would correspond to a phase difference of π; for instance, two signals with phase angles of π/12 and 11π/12, respectively, would only be separated by a phase difference of π/6.

**Figure 4:**
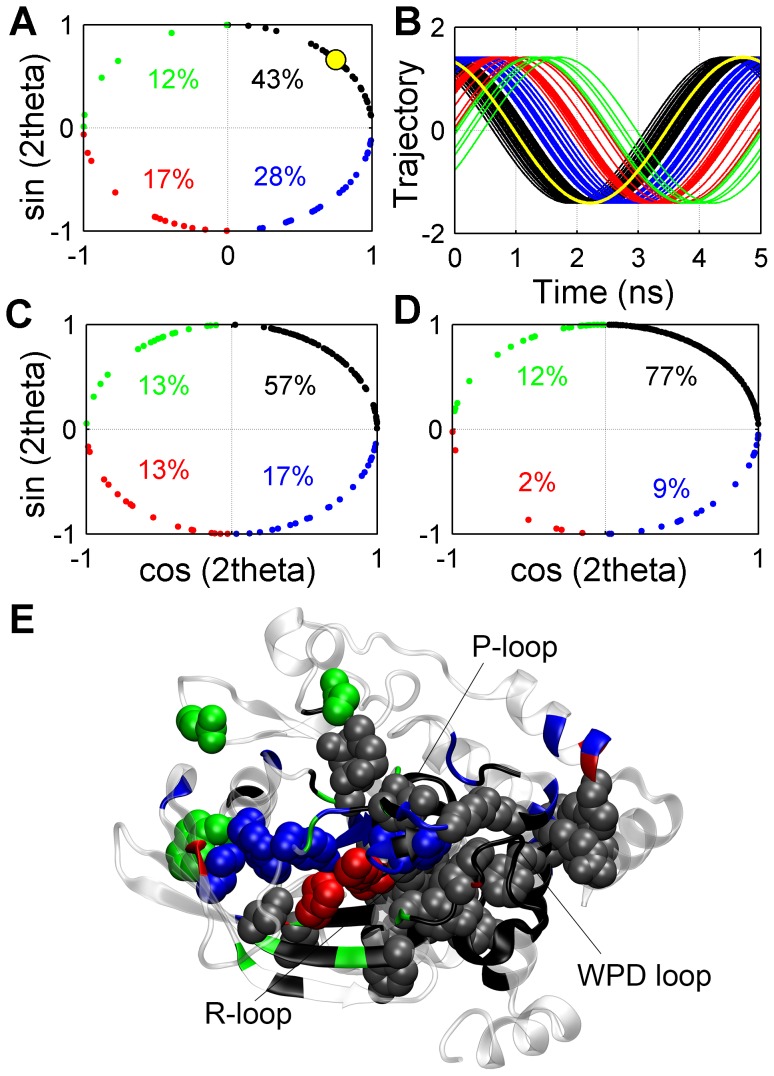
Clustering the perturbed atomic variables with respect to their phase angles. (A) Phase angles of perturbed side-chain dihedral angles on cos(2θ)-sin(2θ) plane. (B) Normalized reconstructed trajectories of the perturbed side-chain dihedral angles. Phase angles of the perturbed (C) backbone dihedral angles and (D) C_α_ displacements on cos(2θ)-sin(2θ) plane. Clusters are numbered in clockwise direction, starting from the first cluster on the upper right quadrant, and colored black, blue, red and green, respectively. On each quadrant of cos-sin planes, percentages of perturbed atomic variables in the corresponding cluster are shown. (E) Residues with perturbed side-chain and backbone dihedral angles are mapped on PTP1B, and colored with respect to their cluster numbers, except for the first cluster, which are colored gray instead of black for easier visualization. Perturbed side-chains are shown with vdW spheres, scaled by 0.8×vdW radii, and perturbed backbone dihedral angles are shown on the ribbon backbone. Phase angle and reconstructed trajectory of Trp179 χ2 dihedral angle, representative of the conformation transition of the WPD loop, are shown in yellow in (A) and (B), respectively.

Perturbed polar interactions are not included in this analysis, since in-phase fluctuations of a pair of atoms may yield out-of-phase interatomic distance fluctuations (Figure S10), thus giving misleading results in clustering. While phase angles of the perturbed side-chain and backbone dihedral angles are distributed all over the unit circle (Figure 4A,C), they are mostly concentrated in the upper right quadrant (0<*θ*<π/4). Lack of well-defined separate clusters led us to classify phase angles by dividing the unit circle into bins of equal intervals. Unit circle was divided into four, taking each quadrant as a separate cluster, i.e. range of phase angles in a single cluster was taken to be π/4. Figure 4B shows the normalized reconstructed trajectories of perturbed side-chain dihedral angles, colored with respect to their clusters in two-dimensional phase-angle plane. There is a distinguishable phase difference between the trajectories of the first and second clusters, which are most populated clusters. Clustering the phase angles of C_α_ displacements (Figure 4D) shows that the first cluster comprises 77% of residues, which span all over the protein (figure not shown). A higher resolution picture of the signaling network is obtained via coloring the residues with respect to their cluster numbers on the PTP1B structure (Figure 4E). Residues comprising the first cluster (gray vdW spheres) spread over PTP1B, from α2′ to L4, and particularly concentrated on the WPD loop and the regions on the opposite side of R-loop. The second cluster residues (blue vdW spheres) mostly reside on the R-loop and β-strands. The third and fourth clusters mainly consist of two small sets of residues, making nonbonded contacts on the other side of PTP1B. Following is a more detailed examination of each cluster of atomic variables.

**Figure 5:**
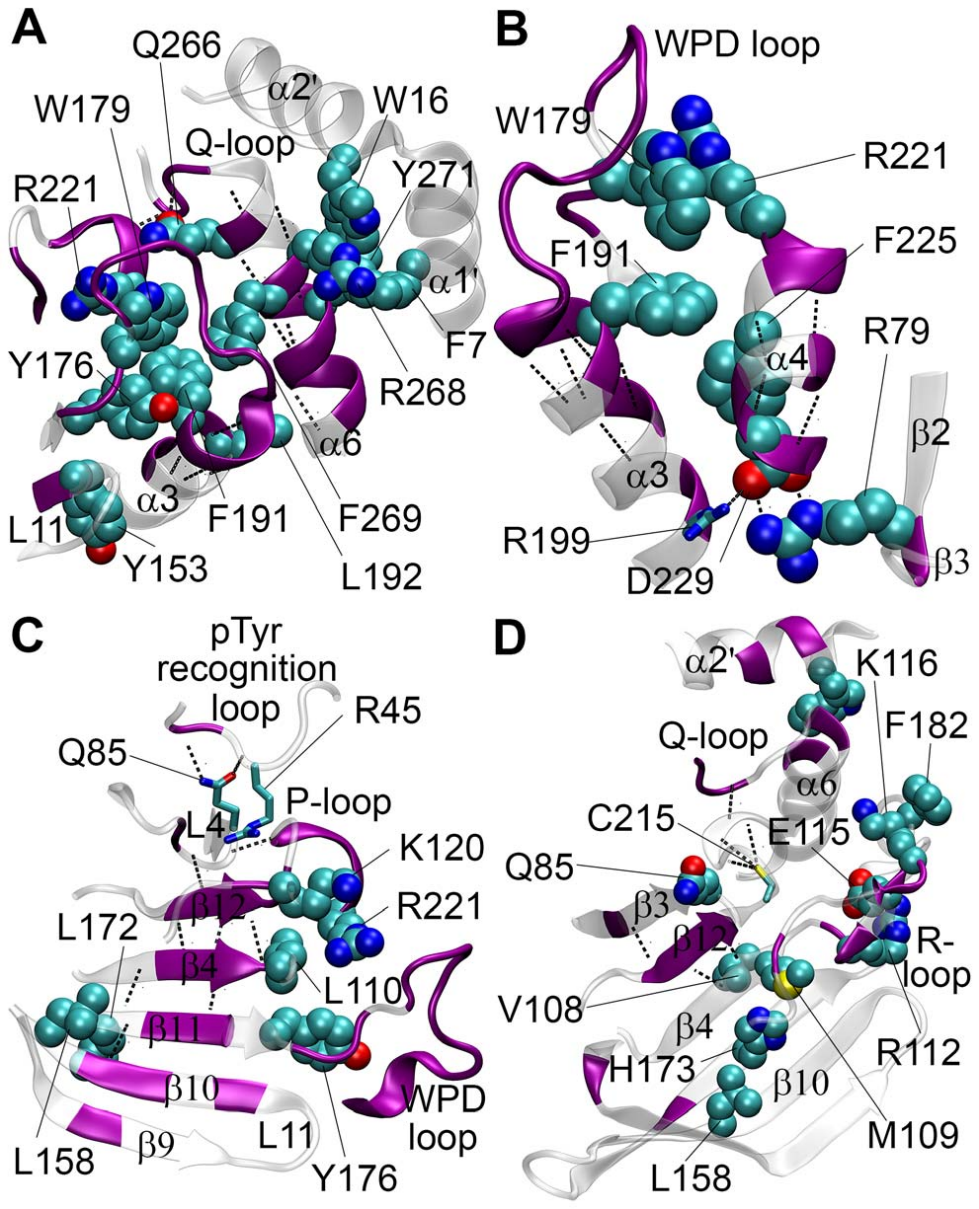
Atomic representations of in-phase fluctuating residues in the first two clusters of atomic variables. Residues with perturbed side-chains in the first cluster contributing to the interactions between (A) WPD loop, L11, α3, P-loop, Q-loop, α6, α1′ and α2′; (B) WPD loop, α3, α4, β2 and β3; (C) WPD loop, pTyr recognition loop, L4, β4, β9, β10, β11, β12 and P-loop. (D) Residues with perturbed side-chains in the second cluster. In all the figures, residues with perturbed side-chains are colored with respect their atoms (nitrogen in blue, oxygen in red, carbon in gray), residues with perturbed backbone dihedral angles are colored in purple, and perturbed H-bonds are shown with black dashed lines. Perturbed side-chains participating in hydrophobic and polar interactions are shown in vdW spheres, with 0.7×vdW radii for easier visualization, and licorice representation, respectively.

The first cluster mostly comprises atomic variables associated with the conformation transition of WPD loop. Signals propagate to outer regions of PTP1B on both sides of WPD loop and to the protein core, so each region located at different sites on PTP1B was investigated separately as a subgroup of the first cluster for easier analysis of their spatial organization. The first subgroup of residues is located in the vicinity of WPD loop extending to its right side (Figure 5A). In-phase fluctuating side-chains of Tyr153 on L11, Tyr176, Trp179 on the WPD loop, Phe191 and Leu192 on α3, Arg221 on P-loop, Gln266 on Q-loop and Phe269 on α6 form an extensive network of hydrophobic interactions, suggesting a plausible mechanism for the concerted perturbations in the dihedral angles of WPD loop, α3, P-loop and Q-loop and α6 (Table 3). These residues (except Phe191) have high identity among human PTP domains [61], while significance of Phe191 and Leu192 in allosteric inhibition and Tyr153 in WPD loop dynamics was recognized in previous studies [48], [52]. Side-chain conformation of Arg221 is a determinant of WPD loop conformation [45] and Gln266 is known to make subtle conformational changes upon repositioning of active waters during WPD loop transition [46], [53]; additionally, significance of both residues in catalysis has been confirmed by mutational studies [62], [63]. Therefore, structural and functional importance of the perturbed residues in the vicinity of WPD loop recognized by experimental studies supports the reliability of our method. Side-chains of Phe7 and Trp16 on α1′–α2′, Arg268 and Tyr271 on α6 made in-phase fluctuations with WPD loop motions, extending the network of hydrophobic interactions. Concerted motion of these side-chains gives a plausible explanation of how C_α_ atoms on α1′–α2′ were perturbed at the same phase angle with WPD loop transition (figure not shown).

**Table 3:** Clustering of residues with respect to phase angles of reconstructed side-chain and backbone dihedral angle trajectories.

Cluster no.	Residues with perturbed side-chain dihedral angles	Structural elements with perturbed backbone dihedral angles
1	Phe7, Trp16, Arg45 (a)(100%), Arg79, Leu110, Lys116, Lys120 (80%), Tyr153 (60%), Leu158, Leu172, Tyr176 (100%), Trp179 (100%), Phe191, Leu192 (80%), Arg221 (100%), Phe225 (40%), Asp229 (80%), Gln266 (90%), Arg268, Phe269 (80%), Tyr271	WPD loop, P-loop, Q-loop, α3, α4, α6, β4, β10, β11
2	Trp16, Gln85, Val108 (80%), Met109 (100%), Arg112, Met114, Glu115 (100%), Lys116 (60%), Gly117 , Lys120 (80%), Leu158, His173, Phe182, Cys215 (100%)	R-loop, Q-loop, β3, β4, β12, α2′, α6
3	Trp96 (100%), Asn111, Tyr124 (100%), Leu160 (40%), Asp181 (80%), Phe182	R-loop, P-loop, L8
4	Asn44 (90%), Gln61, Trp100 (90%), Leu160 (40%)	R-loop, WPD loop, P-loop, β9, β10, β11, α3

(a) Percentage in the parenthesis represents the amino acid identity among 37 human PTP domains [61].

The second subgroup of atomic variables plays a role in signal transduction to the core of PTP1B (Figure 5B). Concerted motion of Phe191 and Phe225, which make hydrophobic interactions, was coupled to fluctuations of Asp229 and Arg79. It was also observed that salt bridges between Arg199, Arg79 and Asp229 were perturbed, indicating a partial contribution of polar interacting side-chains to signal propagation. The last subgroup lies on the left side of WPD loop (Figure 5C) in the vicinity of R-loop. Side-chains of Lys116 and Lys120 on the R-loop and backbone of Cys121 make nonpolar contacts with pTyr recognition loop, L4 and P-loop. Side-chain of Arg45, which formed H-bonds with the backbone oxygens of Gly86 and Pro87, made in-phase fluctuations with WPD loop transitions. Contribution of P-loop to these fluctuations can be deduced from in-phase fluctuations of its backbone and one perturbed H-bond between backbone polar atoms of Ser216 and Gly86. Backbone dihedral angles of β9, β10, β11, β4 and β12, which span PTP1B from its outer rim to its center, made collective fluctuations. There were only two side-chains (Leu158 and Leu172) which contribute to these collective motions, indicating that the perturbation signal is likely to propagate through the β-strands via i) backbone connectivity to the R-loop, WPD loop and P-loop, and ii) H-bonds between backbone atoms.

Most of the perturbed side-chain and backbone dihedral angles in the second cluster belong to residues residing on R-loop (see Table 3). Arg112, Lys116 and Phe182, which play roles in stabilizing WPD loop conformation [45], [53], made collective fluctuations with the backbone of R-loop. Surrounded by Asn111, Arg112, Val113, Cys121 and His175, invariant Met109 participates to a hydrophobic interaction network with His173, Leu158, indicating a plausible signal propagation path from R-loop to the outer β-strands (Figure 5D). Side-chain of the highly conserved Val108 contributed to these in-phase fluctuations, propagating the signal to the interior β-strands (β12 and β3) of PTP1B. It is interesting that the side-chains of Cys215 and Trp16, backbone of α2′ and α6 (Figure 5D) and N-terminal C_α_ atoms of α7 (figure not shown) were also found to be coupled to these fluctuations. One possible mechanism of signal transduction to these regions is via the polar interactions between Cys215 side-chain and backbone amides of P-loop, and the H-bond between the backbone atoms of Ile219 and Ile261. A perturbation in Q-loop may affect α2′ via hydrophobic interactions with Trp16, and α7 through its backbone connectivity to α6, suggesting a coupling mechanism between Q-loop and α7 [52].

The third cluster consists of in-phase perturbed side-chains of residues located in two distant regions; side-chains of Glu115, Asp181 and Phe182 and backbone atoms of R and WPD loops formed the first subgroup, while side-chains of Trp96 on α2, Tyr124 on L8, and Leu160 on β12, making hydrophobic contacts, formed the second subgroup (Figure S11A). The fourth cluster of perturbed side-chains comprises Asn44, Gln61, Trp100, Lys116 and Leu160, and Lys116 and Leu160 make hydrophobic interactions (Figure S11B). Compared to the first three clusters, signaling pathways between the side-chains in the fourth cluster are less evident.

### Effect of forcing function frequency on the magnitude of residue perturbations

**Figure 6:**
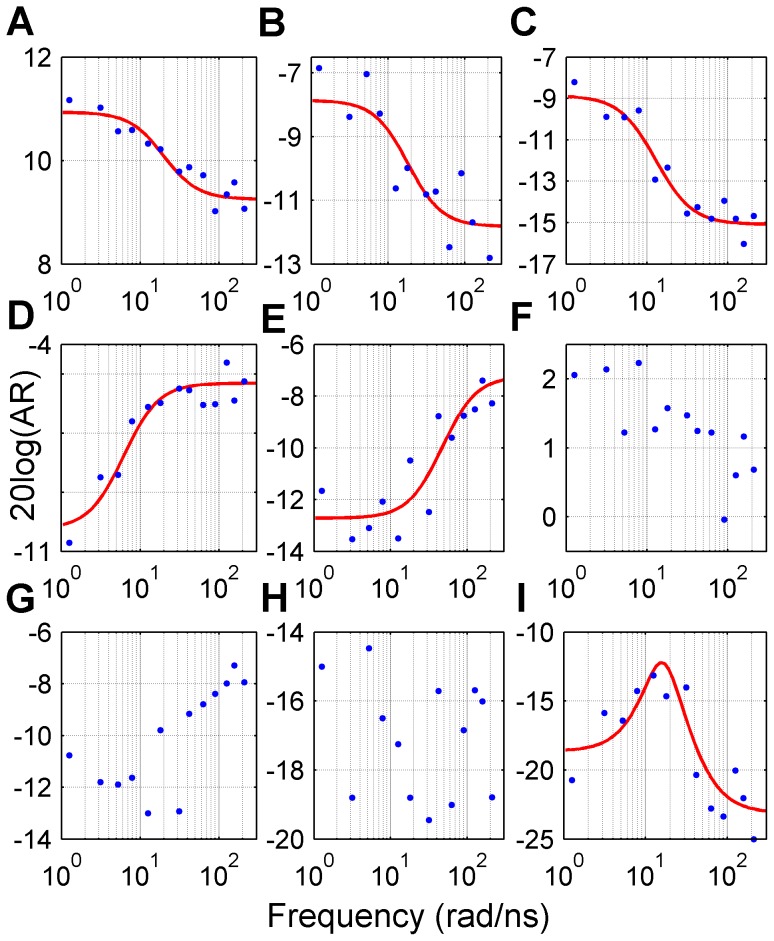
Magnitude Bode plots of C_α_ atomic displacements. Fluctuation amplitudes (blue points) of C_α_ atoms of (A) Asp181, (B) Phe191, (C) Phe269, (D) Lys120, (E) Thr175, (F) Thr178, (G) Tyr176, (H) Pro89 and (I) Cys32 determined at different perturbation frequencies (rad/ns). Red lines represent the fitted lead-lag transfer functions.

In linear systems, input frequency affects the amplitude of output response (see Material and Methods). To examine the response of PTP1B to different frequencies, 13 different TMD simulations were performed at cycling periods of 30 ps (*f_0_* = 33.3 ns^−1^, *w_0_* = 209 rad/ns) to 5 ns (Table S4). Magnitude Bode plots were plotted for each C_α_ atom using the amplitude of fluctuations at the base frequency in each TMD simulation, and stable and minimum-phase systems with a single state were fitted to frequency responses [64]. 60% of C_α_ displacements (Table S5) were found to be well represented by lead-lag transfer functions (Equation 14) and *τ_p_* was found to be greater than *τ_z_* for most of the cases (Figure 6A–C). The 95^th^ percentile of *τ_p_* and *τ_z_* were found to be 220 ps and 210 ps, respectively, giving approximate upper limits for these parameters. Using medians of these parameters, a simple yet informative model of C_α_ dynamics may be as represented as follows:
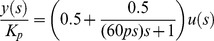
(1)This transfer function states that ∼50% of the final displacement of a C_α_ atom will be realized immediately following the step disturbance, while the rest of the displacement will be in the same direction to its initial perturbations as a first-order relaxation process with a time constant (*τ_p_*) of 60 ps. For 5% of C_α_ atoms, *τ_p_* was found to be smaller than *τ_z_* (Figure 6D,E), indicating that relaxation of these C_α_ atoms would be in the opposite direction to their initial response. The latter response was observed mostly in R-loop residues (Figure S12). About 28% of C_α_ atoms, though showing monotonic trends in their Bode plots, could not be satisfactorily modeled using a single state (Figure 6F,G), while the rest of the C_α_ atoms showed either no trend, or second-order characteristic in their Bode plots (Figure 6H,I).

## Discussion

Allostery has been suggested to be a common feature of all proteins [14]. To have a better understanding of allostery, one must discern intraprotein signal propagation mechanisms. Previous studies have shown that intraprotein signals may be transduced via backbone connectivity, networks formed by side-chains and H-bonds [65]–[67]. In the current study, a simple yet efficient tool is developed to identify signal transduction pathways in proteins. Local periodic perturbations in the form of TMD potentials were given to R-loop and WDP loop, which assumed two distinct conformations in the crystal structures of the free and ligand bound states of PTP1B, and the resulting trajectories of atomic variables were analyzed by statistical tools. As opposed to simplified networks representations [35]–[38], implicit solvent models [31], [34], [35] and low temperature simulations [30], which may cause superfluous increase in signal to noise ratio, MD simulations were performed with explicit solvent at 300 K and without any backbone constraints. Direction and amplitude of applied perturbations were predetermined from functional local conformational changes seen in crystal structures, hence the resulting protein response may be related to function. Using power at the fundamental frequency of C_α_ atoms, conformational transitions of regions on which TMD potential was not directly applied were found to be consistent with those from crystal structures. Spectral densities of atomic variables were observed to vary as 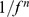 for a wide frequency range, and the linearity of log-log plot of spectral density was exploited to determine the significantly perturbed atomic variables. In the literature, 1/*f* spectrum of fluctuations was observed in resistance of metals [68], and also in protein dynamics [69], [70], and anomalous diffusion was suggested to be one of the mechanisms which could explain this behavior [71]. In the current study, the first ∼10 frequency components showed significant deviations from 1/*f* spectrum (see Figure 1B,S3). This may be a consequence of nonstationary and diffusive character of protein dynamics being manifested through low frequencies [72]. Hence, number of TMD cycles should be sufficiently large (>10) during the whole simulation to prevent random diffusional motions from dominating the base frequency.

Difficulty of constructing signaling pathways using the large number of perturbed C_α_ atoms made it necessary to employ the suggested method on backbone and side-chain dihedral angles and distances between H-bonding pair of atoms. Various studies focused on these atomic variables separately to determine residue couplings in proteins [35], [66], [67], [73], while correlation between backbone and side-chain conformations and H-bonding networks was also recognized [74], [75]. Mapping perturbed atomic variables on the structure of PTP1B showed that these variables were concentrated around the active site, particularly in the vicinity of WPD and P loops, diffusing to the outer regions, corroborating the significance of active and ligand binding sites in protein structure networks [25]. It is important to note that conventional tools may fail in identifying correlated motions of side-chains due to transitions between rotamers. Rotamer transitions inflate the lowest frequency components of dihedral angle trajectories, but Fourier coefficients will decay as frequency increases. Hence, perturbations at medium frequencies, higher than those that are substantially affected by the rotamer transitions, isolate side-chain responses to the local disturbance at the base frequency.

Mapping the perturbed side-chain and backbone dihedral angles on PTP1B structure showed that it was possible to identify interaction networks mainly by examining the nonbonded contacts between the perturbed side-chains, while perturbed backbone conformations and H-bonds made subtle contributions to identification of the networks. In-phase fluctuations of backbone dihedral angles of α3, α4, and α6 and the surrounding side-chains provide evidence for ns-correlation between these atomic variables [75] and cooperation of backbone connectivity and nonbonded interactions in signal transduction. In future applications on other proteins and perturbation sites, different combinations of variables, i.e. displacements of C_α_ atoms and dihedral angles of side-chains, may give better results depending on protein structure and signaling routes. In the current study, clustering was achieved by dividing the phase angles into four equal bins of π/4. Since the base frequency should not be higher than ∼0.8 ns^−1^, there may be >100 ps difference in the fluctuations of the atomic variables within the same cluster. One may decrease the bin width of phase angles for identification of residue couplings in higher resolution. Alternatively, unsupervised clustering methods may be utilized depending on the distribution of phase angles. For instance, employing K-means clustering [76] on the phase angles did not change the first two clusters, hence the current clustering method was unaltered.

The first cluster of atomic variables represents the protein fluctuations coupled with the WDP loop, while the second and third clusters may be significant in maintaining the coupled motions of the β-strands in the core of PTP1B with the R-loop, Q-loop and α7. Abundance of hydrophobic interactions between perturbed side-chains corroborates the view that clusters of hydrophobic side-chains, particularly bulky aromatics [35], around the active site may be dynamically correlated and functionally important [73]. A smaller number of side-chains making polar interactions are also identified as parts of residue interaction network [66]; for instance, salt bridge between Asp229 and Arg79 is likely to play a role in transducing the signal from the WPD loop to the core of PTP1B. Most of the perturbed polar interactions consist of H-bonds formed between backbone atoms, playing roles in signal transduction along helices and between β-strands. Many perturbed residues are found to be moderately to highly conserved among human PTP domains (Table 3), suggesting that conserved residues may be important in coupled motions and signal transduction [77]. Here, we hypothesize that residues on the identified interaction networks are significant in signal propagation from WPD loop to the rest of the protein, but does that compel a communication in the reverse pathway also? Allosteric site in PTP1B comprises α3, α6 and α7 [48], and the current study shows that side-chain fluctuations of residues on the N-terminus of α3–α6 and C_α_ atoms on the N-terminus of α7 are coupled with WPD and R-loop fluctuations, respectively. Communication in reverse pathways was recognized previously [30], [31], hence residues proposed to contribute to signal transduction in the current study may particularly be helpful in guiding and interpreting future experimental studies on allosteric inhibition of PTP1B, a topic of gaining recent importance [78].

Traditionally in linear systems theory, phase angle between two signals is interpreted as time delay, and this interpretation was adopted in a previous study to determine the response times between different residues [31]. We, on the other hand, suggest that this interpretation may be incorrect when atomic variables are taken into consideration. R-loop fluctuations may be given as a representative example to clarify this issue. Although difference in R-loop conformations between WPD_open_ and WPD_closed_ crystal structures is not significant, importance of R-loop mobility was recognized previously for WPD loop transitions [46], [79]. In our analysis, most of the R-loop backbone and side-chain dihedral angles was in the second and third cluster, i.e. the phase difference between the WPD loop and R-loop residues was π/4-π/2. This result is consistent with experimental results, i.e. net displacement of most of R-loop residues between the beginning and end of WPD loop transition is small, whereas R-loop residues may fluctuate significantly during the transition. An idealized case of WPD loop and R-loop fluctuations with a phase angle difference of π/2 may be visualized as follows: As WPD loop moves to its closed conformation, R-loop initially moves away but then return to its initial conformation. We should nevertheless warn the reader about a potential artifact in TMD technique. Recently, it was suggested that TMD related techniques may be biased in the order of conformational transitions, i.e. large-scale changes are likely to occur earlier than small-scale changes [80]. It is possible that this phenomenon may affect the phase angles of atomic variables in our study and requires attention in the future studies.

Bode plots of most of the C_α_ atoms showed low frequency asymptotes, indicating that fluctuations of most of the C_α_ atoms would be significantly attenuated at frequencies higher than a specified value. In previous studies, perturbations were frequently employed at ps scale periods [30], [31], but effect of perturbation frequency on the protein response has not been investigated thoroughly. Here, we show that perturbation frequencies should be lower than the breakpoint frequency (*w_c_*) for the perturbation signal propagate effectively through the protein. Approximate transfer functions of C_α_ displacements were constructed using magnitude Bode plots, and the largest time constants of C_α_ displacements were found to be ∼200 ps, making *w_c_* equal to ∼5 rad/ns (*f_c_* = ∼0.8 ns^−1^). Hence, perturbations with frequencies higher than 0.8 ns^−1^ would lead to dampening of transmitted periodic signal, and the observed coupled motions would not be a faithful representation of the collective response of the protein. Application of low frequency perturbations is also important in increasing the contribution of side-chains to propagation of perturbation signal [33].

Previously, Markovian transmission models were utilized to examine and classify dynamic responses of residues to initial perturbations [38]. Though the motivation, i.e. to elucidate time-dependent dynamics of residues, is similar to that in our study, the employed methods vary significantly. Time-domain response of residues to initial perturbations may be informative in the presence of negligible noise, e.g. network models, but in the more realistic environment of MD simulations with explicit water, frequency response analysis is more convenient to extract time-dependent dynamics. In the current study, response of C_α_ atoms can be classified into two main groups based on their transfer functions. Evenly distributed over PTP1B, the first group consists of C_α_ atoms with initial responses in the same direction with their final displacement upon perturbation. Most of the C_α_ atoms in the second group reside on R-loop and the preceding β4, and the initial responses of these atoms are in the opposite direction to their final displacement. This model complements the role of R-loop dynamics in WPD loop conformational transition, discussed above. R-loop residues move away from their equilibrium positions initially, but tend to return to their initial positions as WPD loop transition proceeds. One should also be cautious in the interpretation of the lead element, which suggests that C_α_ atoms should give an immediate response to local perturbations. While it is physically impossible for the perturbation signal to be transduced immediately, it takes less than ∼10 ps for a local perturbation signal to be propagated through the whole protein at a speed of 5 Å.ps^−1^ to 14 Å.ps^−1^, as suggested in the literature [30], [31]. Considering that period of the fastest local perturbation was 30 ps in the current study, the instantaneous response suggested by the lead element should be interpreted as a response within the first ∼10 ps.

The current study has also yielded some interesting questions. Are higher harmonics observed in the frequency spectra a consequence of the rectangular form of input function, or nonlinear nature of the protein machine [81]? Are different Bode plots characteristics of residues related to their functional roles? It is expected that future applications of frequency response techniques on other proteins will not only enrich our understanding of allostery, but also improve the current method by illuminating these and other issues.

## Materials and Methods

### Equilibrium Molecular Dynamics (EMD) simulations

Initial atomic coordinates for WDP_open_ and WDP_closed_ structures and crystal structure waters within 6 Å of PTP1B were obtained from PDB [82] with PDB IDs 2F6F [49] and 1SUG [46], respectively. Cys215 was taken in thiolate form [83], while Asp181 was protonated [84]. In the crystal structure of 2F6F, residues −3 to 0 were truncated and Phe295 was back mutated to Ser295. In 1SUG crystal structure, Leu299 was removed and Met1 was added to this structure using 2F6F structure as a template. Missing side-chain and hydrogen coordinates were estimated using psfgen package of VMD [85]. TIP3 waters were added within a layer of 10 Å of the protein in a rectangular box of 79.6×80.5×69.1 Å, and the system was neutralized by adding sodium and chloride ions. Particle mesh Ewald method [86] and a non-bonded cutoff of 12 Å were employed on the system containing 4830 protein atoms and 12000 water molecules. NAMD [87] program was used with the CHARMM27 forcefield with cmap correction [88], and thiolate CHARMM forcefield parameters were taken from Foloppe et al. [89]. Minimization of 3000 steps was followed by a gradual heating to 300 K at an integration time step of 1 fs. Equilibrium simulations were performed at a constant temperature of 300 K using a damping coefficient of 5 ps^−1^ for Langevin temperature control, and at a constant pressure of 1 atm using 100 fs and 50 fs as the oscillation period and the damping time scale, respectively, for Nose-Hoover Langevin piston pressure. Two equilibrium MD simulations of 40 ns length in both conformations were performed, and representative snapshots were taken as target structures to be used in TMD simulations. An additional EMD simulation of 80 ns length in WPD_open_ conformation was performed, as a reference equilibrium simulation to be compared with TMD simulation of the same length.

### Restrained Targeted Molecular Dynamics (TMD) simulations

A subset of atoms is driven to a target conformation in restrained TMD simulations with holonomic constraint [90]. TMD force on each atom in the subset is computed by the gradient of the following potential:
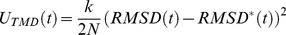
(2)where RMSD(*t*) is the RMSD between the current and target coordinates, and RMSD^*^(*t*) is a positive scalar linearly decreasing from the value of the initial RMSD between the first and target structures to zero. In the current study, TMD potential was initially applied on WPD loop atoms only, but side-chain of Glu115 on R-loop was seen to hinder the closure of WPD loop [46], [79], so TMD potential was extended to include R-loop atoms also. Smallest spring constant *k* rendering periodic WPD loop transitions [91] was found to be 3000 kcal·mol^−1^·Å^−2^ by trial and error. Analysis of the WPD loop trajectory on the reduced PC plane and repeating the analysis using TMD simulations with a smaller *k* value showed the robustness of the current results (Text S5 and Figure S13, S14, S15, S16, S17). Target structures were altered between the equilibrated structures of PTP1B in WPD_open_ and WPD_closed_ conformations. A set of TMD simulations was performed to elucidate the effect of perturbation frequency on the response of atomic variables (see Table S4).

### Principal Components Analysis (PCA)

PCA is a conventional statistical tool used in dimension reduction of multivariable data [92], hence a convenient tool to handle the high number of degrees of freedom in proteins [93]. In traditional application of PCA, snapshots of C_α_ coordinates are placed in succeeding rows of a trajectory matrix *X*, in which each row consists of 3*N* variables (coordinates), with *N* equal to number of C_α_ atoms. Spectral decomposition of the covariance matrix (*C*) of *X* gives the eigenvectors matrix *P* and eigenvalues matrix Λ, whose diagonal elements *λ*_i_ are the variances of the collective coordinates on the eigenvector *p*_i_ (principal axes).
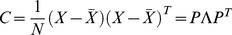
(3)Here, indices of eigenvectors are ranked in decreasing order of eigenvalues, hence the first few eigenvectors are assumed to capture the significant variation of the data. Collective coordinates (*t_i_*) in principal component (PC) subspace can be determined via projecting the mean-centered trajectory matrix (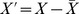) on a subset of eigenvectors, such as the essential subspace (*P_i_*):
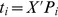
(4)Projecting the collective coordinates back to the original variable space removes the orthogonal components to the reduced PC plane, practically acting as a filter.
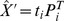
(5)


### Comparison of predicted and experimental residue displacements

Overlap (*I*) and correlation coefficient are two metrics used to compare the residue displacements estimated via PCA with those obtained from crystal structures [94]. Taking *p_ij_* as the contribution of the *i* ^th^ variable on the *j* ^th^ eigenvector from PCA, and *d_i_* as the displacement of *i* ^th^ variable (Cartesian coordinates of C_α_ atoms) between two crystal structures, overlap of *j* ^th^ eigenvector is defined as follows:
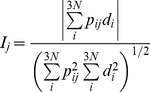
(6)Overlap measures the similarity of the residue displacement directions determined by the experimental and computational methods. Correlation coefficient, on the other hand, measures the similarity of the overall pattern of amplitudes of displacements:
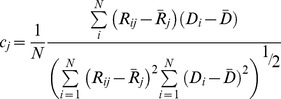
(7)Here, *R_ij_* and *D_i_* are the amplitudes of displacement of the *i* ^th^ C_α_ atom determined by the *j* ^th^ eigenvector and crystal structures, respectively, while 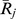 and 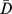 represent the average displacements in each set. In the current study, a single eigenvector (*j* = 1) is used in results obtained from TMD simulations, thus the second subscript in *p* and *R* vectors and the single subscript in *I* and *c* values are omitted. For the EMD simulation, eigenvectors are denoted by *e_j_*, in which the subscript *j* represents the index of the eigenvector.

### Discrete Fourier Transform (DFT)

Discrete Fourier Transform (DFT) is the Fourier analysis applied to discrete periodic data to decompose the signal into harmonic sinusoidal components. For a discrete *N*-periodic time series *x_n_*, *n* = 0, 1,…, *N*-1, DFT is computed as
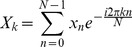
(8)Here, *X_k_* is a complex number representing both the amplitude and phase of the *k* ^th^ sinusoidal component. The original series *x_n_* can be recovered using inverse DFT (IDFT) as
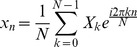
(9)Parseval's relation states that energy of a signal (*E*), which is equal to the squared sum of signal values in time domain, can be also obtained by the squared sum of the magnitude of the DFT coefficients [54].
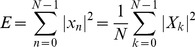
(10)When the signal is mean-centered, i.e. deviation of the trajectory from its mean value, power of the signal is equivalent to its MSF:
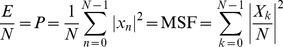
(11)Hence, contribution of *k* ^th^ periodic component to MSF is equal to 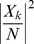 (Text S6).

### Laplace domain representation of linear systems

Analysis of linear systems may be difficult in time domain representation. A more convenient representation is achieved via transfer functions (*G_p_*) in Laplace domain. Transfer functions contain all the dynamic and steady state information about a system, thus classification of systems is easier in Laplace Domain. For instance, a first order lag system is represented in time domain with the following representation:
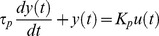
(12)where *τ_p_* and *K_p_* are time constant and steady state gain, respectively; *u* and *y* are the input and output of the system, respectively. The transfer function representation of the same system is shown as follows:
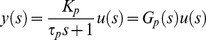
(13)where *s* is the complex variable in Laplace domain [95]. In the current study, a lead element, which increases the speed of response, is added to the numerator of the transfer function, thus the resulting system is a lead-lag system:
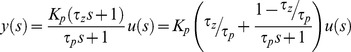
(14)In this equation, *τ_z_* represents the time constant of the lead element, and response characteristics depends on *τ_p_* and *τ_z_*/*τ_p_* ratio (Text S7).

### Frequency response of linear systems

Frequency response analysis is a powerful tool particularly used in process control systems. Frequency response, basically, is the steadystate response of a system subject to a sustained sinusoidal input [96]. If the input to a linear system, shown with *G_p_*(*s*) in Laplace domain, is a sine wave with frequency *f_o_*, then the output at steady state (*y_ss_*) will also be a sine wave at the same frequency but at a different amplitude and a phase lag (*θ*).
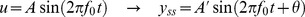
(15)Bode plot is a useful tool in analyzing how the amplitude ratio (AR), which is the ratio of the output amplitude (*A′*) to input amplitude (*A*), changes with respect to different input signal frequencies. In magnitude Bode plot, logarithmic scales are used, and ordinate is plotted using 20log AR [95]. Many systems can be described by a series of first-order lags, thus frequency response of a first order system requires a special attention. A first order system has a flat low-frequency and a decreasing high-frequency asymptote, which intersect at the breakpoint frequency of 1/*τ_p_* (Figure S18). This process acts a low-pass filter, which passes frequencies below the breakpoint frequency, and attenuates frequencies above the breakpoint frequency.

## Supporting Information

Figure S1 **Crystal structures of PTP1B in WPD_open_ and WPD_closed_ conformations.** WPD_open_ (PDB ID: 2F6F) and WPD_closed_ (PDB ID: 1SUG) structures are shown with blue and red, respectively. Open and closed conformations of the WPD loop are shown in ice blue and green, respectively.
(PDF)

Figure S2 **RMSD of various structural elements of PTP1B in EMD and TMD_1_ simulations from crystal structures.** (A) RMSD of PTP1B in MD (black) and TMD_1_ (blue) simulations from the WPD_open_ crystal structure (PDB ID: 2F6F). (B) RMSD of the WPD loop in EMD simulation from WPD_open_ (black) and WPD_closed_ (gray, PDB ID: 1SUG) crystal structures. (C) RMSD of the WPD loop in TMD_1_ simulation from WPD_open_ (dark blue) and WPD_closed_ (light blue) crystal structures. (D) RMSD of α7 (residues 281 to 298) in EMD simulation from WPD_open_ (black) and WPD_closed_ (gray) crystal structures. RMSD of the same region in TMD_1_ simulation from WPD_open_ (dark blue) and WPD_closed_ (light blue) crystal structures. Disordered nature of α7 is confirmed by EMD and TMD simulations.
(PDF)

Figure S3 **Comparison of power spectral density functions of EMD and TMD_1_ simulations.** Power spectral density per residue (or residue-averaged MSF) for (A) WPD loop, and (B) residues on which TMD potential was not directly applied. Frequency components of EMD and TMD_1_ simulations are represented with black and blue solid lines, respectively. Gray and yellow dashed lines represent the least-squares lines fit to EMD and TMD_1_ data, respectively. Base frequency and the upper harmonics (peaks) are more clearly seen in the C_α_ atomic trajectory spectrum of the WPD loop. Existence of peaks in the power spectrum of regions on which TMD potential was not directly applied shows that effects of local disturbance propagated to the rest of the protein.
(PDF)

Figure S4 **Transformation of reconstructed trajectories to reconstructed in-phase trajectories using PCA.** (A) Reconstructed trajectories of three-Cartesian components of WPD loop C_α_ atoms. (B) In-phase components obtained by employing PCA on the trajectories shown in (A). (C) In-phase components obtained by employing PCA on the reconstructed trajectories of C_α_ atoms between residues 2 to 278.
(PDF)

Figure S5 **Comparison of estimated and experimental C_α_ displacement amplitudes.** Amplitudes of C_α_ displacements estimated using reconstructed in-phase trajectories are compared with displacements obtained from the averages of all crystal structures in WPD_open_ and WPD_closed_ conformation listed in Table S1. Estimated and experimental displacements are shown in blue and black, respectively.
(PDF)

Figure S6 **Different conformations of L16 (Asp236 to Ser243) adopted in crystal structures.** Blue and red represent L16_I_ and L16_II_ conformations adopted in WPD_closed_ crystal structures, respectively, while ice blue and purple represents L16_I_ and L16_II_ conformations adopted in WPD_open_ crystal structures, respectively.
(PDF)

Figure S7 **Amplitude of residue displacements predicted from low-frequency TMD simulations.** TMD_1_ (blue), TMD_2_ (green) and TMD_3_ (red) simulations correspond to cycling periods of 5 ns, 2 ns, and 1.2 ns, respectively.
(PDF)

Figure S8
**Effect of sampling interval and perturbation frequency on the frequency response of residues in TMD_1_.** (A) Power spectral density of all residues except α7 sampled at 0.5, 1, 2 and 5 ps intervals. Vertical green dotted lines intersecting the frequency axis at ∼6 ns^−1^ represent the upper frequency limit of power spectrum which is assumed to obey 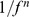 distribution. (B) Percent of perturbed C_α_ atoms identified using different number of TMD simulation cycles ranging from one to 16.(PDF)

Figure S9 **Power spectral density of various perturbed side-chain dihedral angles in the vicinity of WPD loop.** Power component at 0.2 and 0.4 ns^−1^ are denoted by a square and a triangle, respectively.
(PDF)

Figure S10 **Sensitivity of phase angles of interatomic distances to amplitudes of atomic fluctuations.** (A) Two approximately in-phase signals (atomic positions) with a phase difference of π/33 and a unit difference between their amplitudes, and (B) difference (interatomic distance) between the amplitudes of these two signals; new signal is also approximately in-phase with the first two signals. (C) Two approximately in-phase signals with a phase difference of π/33 and 5% difference between their amplitudes, and (D) difference between the amplitudes of these two signals; phase difference between new signal and the first two signals is approximately π/2.
(PDF)

Figure S11 **Atomic representations of in-phase fluctuating residues in the third and fourth clusters of atomic variables.** (A) The third cluster of in-phase fluctuations consists of coupled motions of the backbone of R-loop, side-chains of Asp181, Phe182 on the WPD loop, and Trp96, Tyr124 and Leu160, which make hydrophobic interactions. (B) The fourth cluster of in-phase fluctuations consists of coupled motions of the backbones of β9, β10 and β11 and side-chains of Asn44, Gln61, Trp100 and Leu160. Out of these four residues, the last two make hydrophobic contacts. In both figures, residues with perturbed side-chains are colored with respect their atoms (nitrogen in blue, oxygen in red, carbon in gray), regions of backbones with perturbed dihedral angles are colored in purple, and perturbed H-bonds are shown with black dashed lines.
(PDF)

Figure S12 **Classification of the residues with respect to their frequency responses.** Blue and red colored residues have monotonic decreasing and monotonic increasing frequency responses, respectively. Concave functions may be fitted to the frequency responses of yellow residues, indicating an underdamped behavior. No definite trends in Bode plots of white residues have been observed (see Table S5). Transparent regions represent C_α_ atoms not being perturbed by the TMD potential.
(PDF)

Figure S13 **Trajectory of** ***φ*** **_182_ backbone dihedral angle in TMD_1_ simulation.** Black and maroon dashed lines represent the angles adopted by *φ*_182_ in WPD_open_ and WPD_closed_ crystal structures. Yellow lines represent the trajectory of *φ*_182_ from WPD_open_ to WPD_closed_ conformations, while blue lines represent the trajectory of *φ*_182_ from WPD_closed_ to WPD_open_ conformations.
(PDF)

Figure S14 **WPD loop conformational transition on the reduced PC planes.** Small black dots represent the whole trajectory of WPD loop during TMD_1_ simulation. Pink and green lines represent the WPD loop trajectory during the first loop closing (WPD_open_→WPD_closed_) and the first loop opening (WPD_closed_→WPD_open_), respectively. Blue and red filled circles represent the WPD_open_ and WPD_closed_ crystal structures, respectively. Yellow filled square and purple filled circles connected by gray arrows denote the trajectory of WPD loop in the absence of active water molecules in the WPD_closed_ state. Light gray crosses represent an equilibrium simulation in WPD_closed_ conformation. The numbers in parenthesis on the axis labels denote the percentage of explanation offered by PCA.
(PDF)

Figure S15 **Histograms of the backbone dihedral angles of WPD loop residues.** Blue and red lines represent the averages of the dihedral angles adopted during the first 50 ps and the last 50 ps in the first WPD loop transition.
(PDF)

Figure S16 **Structural analysis of WPD loop in TMD′ simulation.** (A) RMSD of the WPD loop from its conformation in WPD_open_ (dark blue) and WPD_closed_ (light blue) crystal structures. (B) Trajectory of *φ*_182_ dihedral angle. Lines and coloring are identical to those in Figure S13. (C) Histogram of the backbone dihedral angles of Phe182.
(PDF)

Figure S17 **Comparison of WPD loop conformational transitions in TMD simulations with different spring constants on the reduced PC planes.** Projection of WPD loop transitions on (A) PC1–PC2, and (B) PC1–PC3 planes. Black and blue circles represent TMD simulations with spring constant equal to 3000 kcal·mol^−1^·Å^−2^ and 500 kcal·mol^−1^·Å^−2^, respectively. (C) Transition of WPD loop in the first cycle of both simulations on PC1–PC2 plane. Black (TMD_1_) and blue lines (TMD′) represent the WPD loop trajectory during the first loop closing, while red (TMD_1_) and green (TMD′) represent the first loop opening.
(PDF)

Figure S18 **Time and frequency domain responses of first order lag and lead-lag systems.** (A) Step, and (B) frequency responses of a first order lag system. In (B), breakpoint frequency is equal to 1/*τ_p_* = 0.1 rad/s. Unit step responses of lead-lag processes with (C) *τ_z_*/*τ_p_*<1, and (D) *τ_z_*/*τ_p_*>1. Magnitude Bode plots of lead-lag processes with (E) *τ_z_*/*τ_p_*<1, and (F) *τ_z_*/*τ_p_*>1.
(PDF)

Table S1 **PDB IDs of the crystal structures used in the current study.**
(PDF)

Table S2 **Overlap of residue displacements of reconstructed trajectories determined from low frequency TMD simulations.**
(PDF)

Table S3 **Number of perturbed C_α_ atoms determined from low frequency TMD simulations.**
(PDF)

Table S4 **List of TMD simulations performed and analyzed in the current study.**
(PDF)

Table S5 **Classification of magnitude Bode plot data of C_α_ atoms.**
(PDF)

Text S1 **Single eigenvalue representation of collective residue displacements.**
(PDF)

Text S2 **Conformations of L16 adopted in the crystal structures of PTP1B.**
(PDF)

Text S3 **Determination of a single reconstructed trajectory for each C_α_ atom.**
(PDF)

Text S4 **Robustness of the frequency response method with respect of resolution of power spectra.**
(PDF)

Text S5 **Are trajectories produced by TMD simulations realistic?**
(PDF)

Text S6 **Relation between power of a signal and atomic MSF.**
(PDF)

Text S7 **Time and frequency responses of first order lag and lead-lag systems.**
(PDF)
