# Supplementary material for: Frequency Response of a Protein to Local Conformational Perturbations
Source: PLoS Comput Biol. 2013 Sep 26;9(9):e1003238. doi: 10.1371/journal.pcbi.1003238 (PMC3784495; doi:10.1371/journal.pcbi.1003238)
Supplement: Figure S1 — Crystal structures of PTP1B in WPDopen and WPDclosed conformations. WPDopen (PDB ID: 2F6F) and WPDclosed (PDB ID: 1SUG) structures are shown with blue and red, respectively. Open and closed conformations of the WPD loop are shown in ice blue and green, respectively. (PDF) [file pcbi.1003238.s001.pdf]

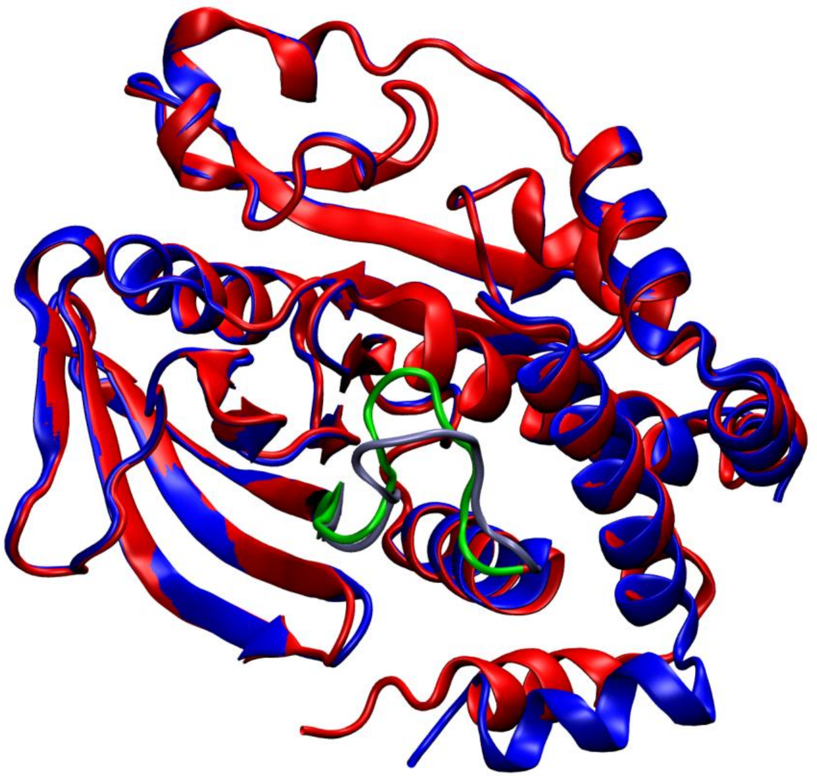

Figure S1. Crystal structures of PTP1B in WPD$_{open}$ and WPD$_{closed}$ conformations. WPD$_{open}$ (PDB ID: 2F6F) and WPD$_{closed}$ (PDB ID: 1SUG) structures are shown with blue and red, respectively. Open and closed conformations of the WPD loop are shown in ice blue and green, respectively.
