# Supplementary material for: Frequency Response of a Protein to Local Conformational Perturbations
Source: PLoS Comput Biol. 2013 Sep 26;9(9):e1003238. doi: 10.1371/journal.pcbi.1003238 (PMC3784495; doi:10.1371/journal.pcbi.1003238)
Supplement: Figure S2 — RMSD of various structural elements of PTP1B in EMD and TMD1 simulations from crystal structures. (A) RMSD of PTP1B in MD (black) and TMD1 (blue) simulations from the WPDopen crystal structure (PDB ID: 2F6F). (B) RMSD of the WPD loop in EMD simulation from WPDopen (black) and WPDclosed (gray, PDB ID: 1SUG) crystal structures. (C) RMSD of the WPD loop in TMD1 simulation from WPDopen (dark blue) and WPDclosed (light blue) crystal structures. (D) RMSD of α7 (residues 281 to 298) in EMD simulation from WPDopen (black) and WPDclosed (gray) crystal structures. RMSD of the same region in TMD1 simulation from WPDopen (dark blue) and WPDclosed (light blue) crystal structures. Disordered nature of α7 is confirmed by EMD and TMD simulations. (PDF) [file pcbi.1003238.s002.pdf]

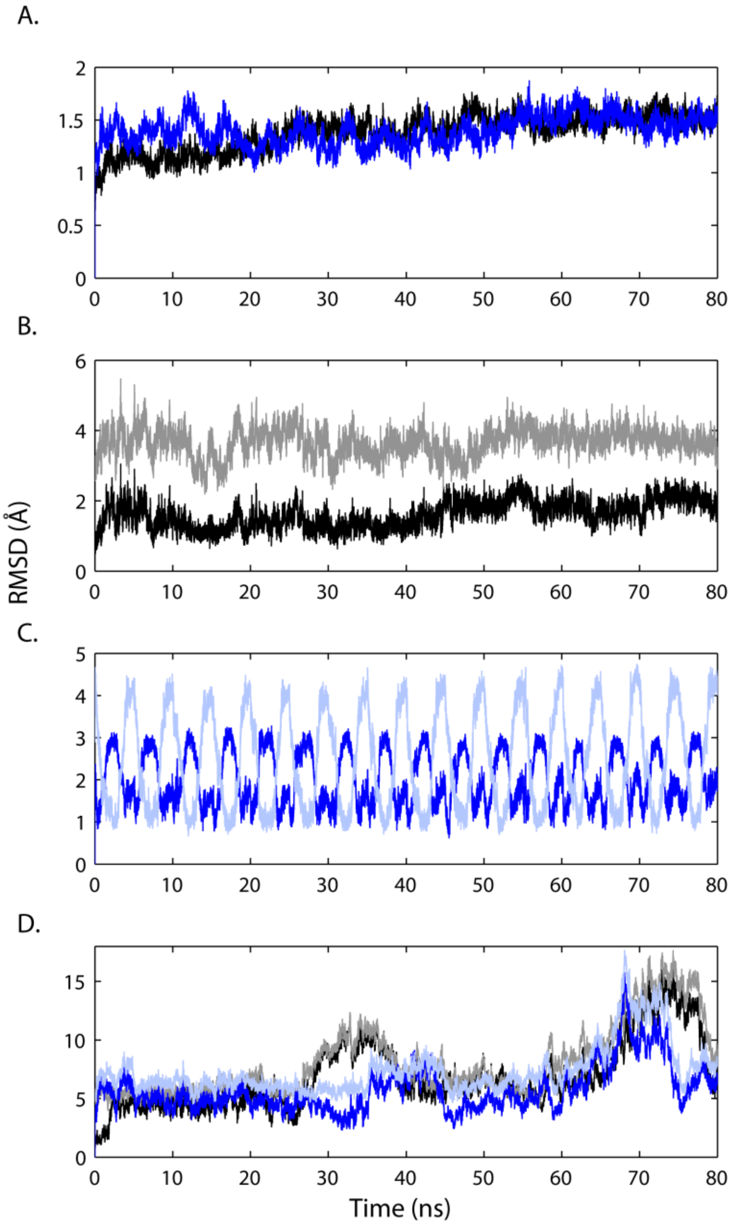

Figure S2. RMSD of various structural elements of PTP1B in EMD and TMD$_1$ simulations from crystal structures. (A) RMSD of PTP1B in MD (black) and TMD$_1$ (blue) simulations from the WPD$_{open}$ crystal structure (PDB ID: 2F6F). (B) RMSD of the WPD loop in EMD simulation from WPD$_{open}$ (black) and WPD$_{closed}$ (gray, PDB ID: 1SUG) crystal structures. (C) RMSD of the WPD loop in TMD$_1$ simulation from WPD$_{open}$ (dark blue) and WPD$_{closed}$ (light blue) crystal structures. (D) RMSD of α7 (residues 281 to 298) in EMD simulation from WPD$_{open}$ (black) and WPD$_{closed}$ (gray) crystal structures. RMSD of the same region in TMD$_1$ simulation from WPD$_{open}$ (dark blue) and WPD$_{closed}$ (light blue) crystal structures. Disordered nature of α7 is confirmed by EMD and TMD simulations.
