# Supplementary material for: Frequency Response of a Protein to Local Conformational Perturbations
Source: PLoS Comput Biol. 2013 Sep 26;9(9):e1003238. doi: 10.1371/journal.pcbi.1003238 (PMC3784495; doi:10.1371/journal.pcbi.1003238)
Supplement: Figure S3 — Comparison of power spectral density functions of EMD and TMD1 simulations. Power spectral density per residue (or residue-averaged MSF) for (A) WPD loop, and (B) residues on which TMD potential was not directly applied. Frequency components of EMD and TMD1 simulations are represented with black and blue solid lines, respectively. Gray and yellow dashed lines represent the least-squares lines fit to EMD and TMD1 data, respectively. Base frequency and the upper harmonics (peaks) are more clearly seen in the Cα atomic trajectory spectrum of the WPD loop. Existence of peaks in the power spectrum of regions on which TMD potential was not directly applied shows that effects of local disturbance propagated to the rest of the protein. (PDF) [file pcbi.1003238.s003.pdf]

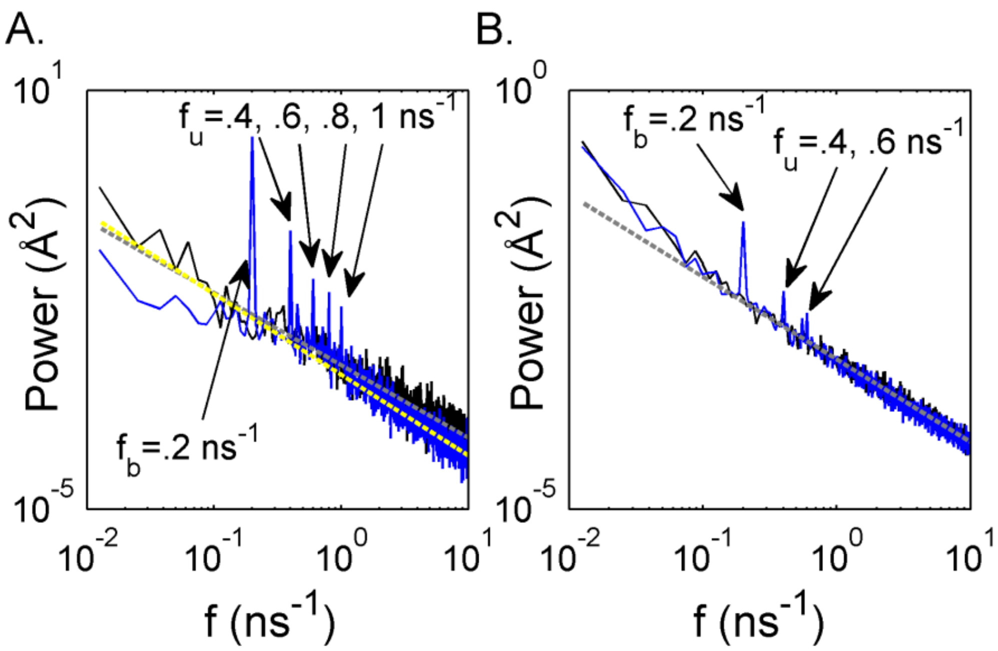

Figure S3. Comparison of power spectral density functions of EMD and TMD_1 simulations. Power spectral density per residue (or residue-averaged MSF) for (A) WPD loop, and (B) residues on which TMD potential was not directly applied. Frequency components of EMD and TMD_1 simulations are represented with black and blue solid lines, respectively. Gray and yellow dashed lines represent the least-squares lines fit to EMD and TMD_1 data, respectively. Base frequency and the upper harmonics (peaks) are more clearly seen in the C_α atomic trajectory spectrum of the WPD loop. Existence of peaks in the power spectrum of regions on which TMD potential was not directly applied shows that effects of local disturbance propagated to the rest of the protein.
