# Supplementary material for: Frequency Response of a Protein to Local Conformational Perturbations
Source: PLoS Comput Biol. 2013 Sep 26;9(9):e1003238. doi: 10.1371/journal.pcbi.1003238 (PMC3784495; doi:10.1371/journal.pcbi.1003238)
Supplement: Figure S4 — Transformation of reconstructed trajectories to reconstructed in-phase trajectories using PCA. (A) Reconstructed trajectories of three-Cartesian components of WPD loop Cα atoms. (B) In-phase components obtained by employing PCA on the trajectories shown in (A). (C) In-phase components obtained by employing PCA on the reconstructed trajectories of Cα atoms between residues 2 to 278. (PDF) [file pcbi.1003238.s004.pdf]

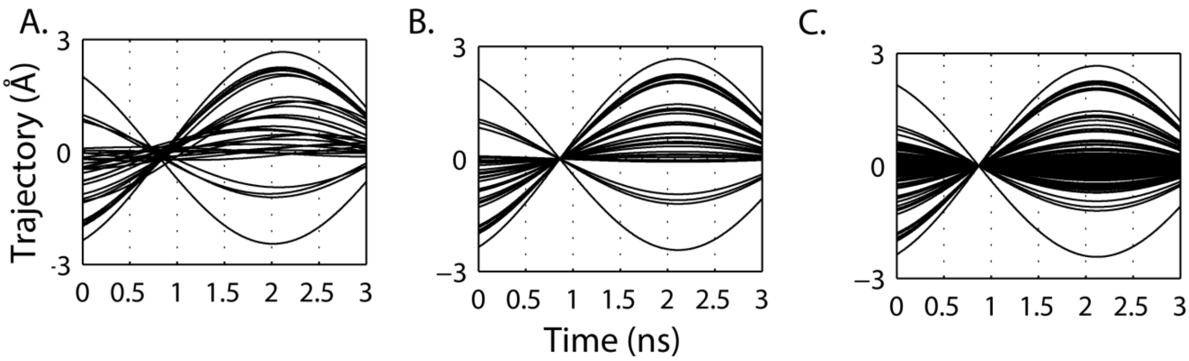

Figure S4. Transformation of reconstructed trajectories to reconstructed in-phase trajectories using PCA. (A) Reconstructed trajectories of three-Cartesian components of WPD loop C_α atoms. (B) In-phase components obtained by employing PCA on the trajectories shown in (A). (C) In-phase components obtained by employing PCA on the reconstructed trajectories of C_α atoms between residues 2 to 278.
