# Supplementary material for: Frequency Response of a Protein to Local Conformational Perturbations
Source: PLoS Comput Biol. 2013 Sep 26;9(9):e1003238. doi: 10.1371/journal.pcbi.1003238 (PMC3784495; doi:10.1371/journal.pcbi.1003238)
Supplement: Figure S5 — Comparison of estimated and experimental Cα displacement amplitudes. Amplitudes of Cα displacements estimated using reconstructed in-phase trajectories are compared with displacements obtained from the averages of all crystal structures in WPDopen and WPDclosed conformation listed in Table S1. Estimated and experimental displacements are shown in blue and black, respectively. (PDF) [file pcbi.1003238.s005.pdf]

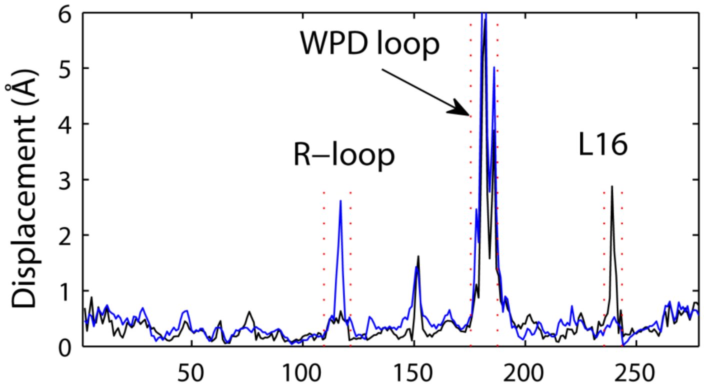

Figure S5. Comparison of estimated and experimental C_α displacement amplitudes. Amplitudes of C_α displacements estimated using reconstructed in-phase trajectories are compared with displacements obtained from the averages of all crystal structures in WPD_{open} and WPD_{closed} conformation listed in Table S1. Estimated and experimental displacements are shown in blue and black, respectively.
