# Supplementary material for: Frequency Response of a Protein to Local Conformational Perturbations
Source: PLoS Comput Biol. 2013 Sep 26;9(9):e1003238. doi: 10.1371/journal.pcbi.1003238 (PMC3784495; doi:10.1371/journal.pcbi.1003238)
Supplement: Figure S6 — Different conformations of L16 (Asp236 to Ser243) adopted in crystal structures. Blue and red represent L16I and L16II conformations adopted in WPDclosed crystal structures, respectively, while ice blue and purple represents L16I and L16II conformations adopted in WPDopen crystal structures, respectively. (PDF) [file pcbi.1003238.s006.pdf]

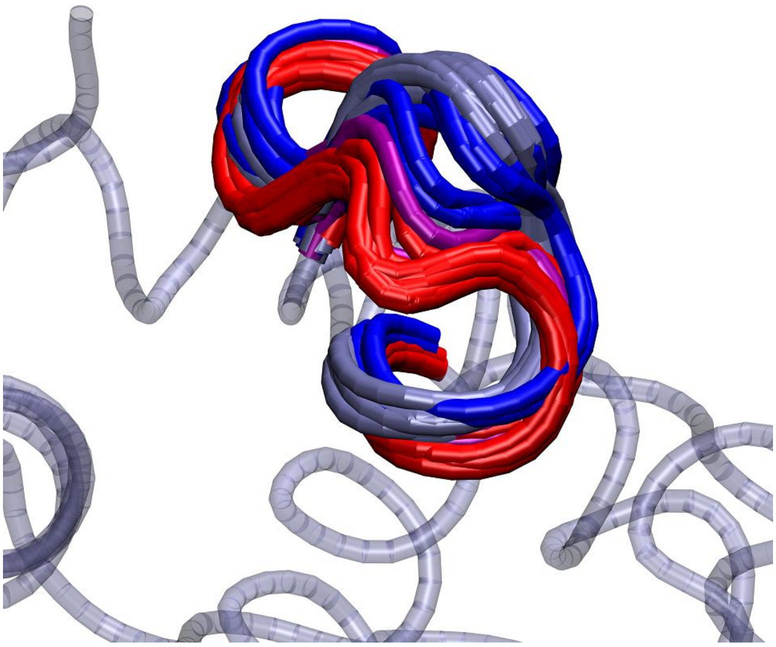

Figure S6. Different conformations of L16 (Asp236 to Ser243) adopted in crystal structures. Blue and red represent $L16_{I}$ and $L16_{II}$ conformations adopted in WPD_{closed} crystal structures, respectively, while ice blue and purple represents $L16_{I}$ and $L16_{II}$ conformations adopted in WPD_{open} crystal structures, respectively.
