# Supplementary material for: Frequency Response of a Protein to Local Conformational Perturbations
Source: PLoS Comput Biol. 2013 Sep 26;9(9):e1003238. doi: 10.1371/journal.pcbi.1003238 (PMC3784495; doi:10.1371/journal.pcbi.1003238)
Supplement: Figure S7 — Amplitude of residue displacements predicted from low-frequency TMD simulations. TMD1 (blue), TMD2 (green) and TMD3 (red) simulations correspond to cycling periods of 5 ns, 2 ns, and 1.2 ns, respectively. (PDF) [file pcbi.1003238.s007.pdf]

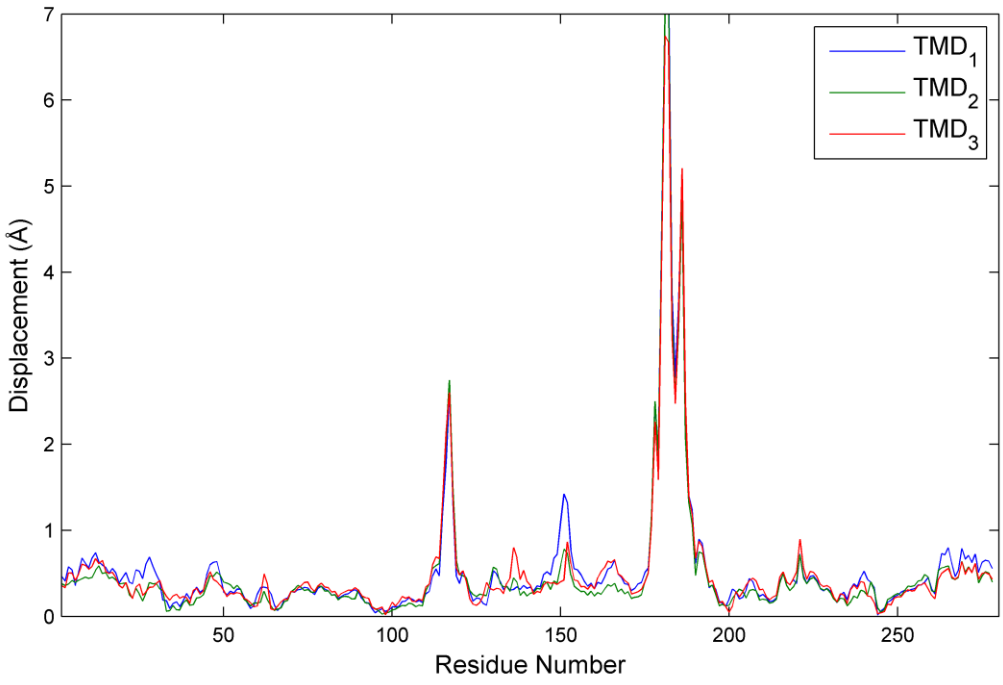

Figure S7. Amplitude of residue displacements predicted from low-frequency TMD simulations. TMD_1 (blue), TMD_2 (green) and TMD_3 (red) simulations correspond to cycling periods of 5 ns, 2 ns, and 1.2 ns, respectively.
