# Supplementary material for: Frequency Response of a Protein to Local Conformational Perturbations
Source: PLoS Comput Biol. 2013 Sep 26;9(9):e1003238. doi: 10.1371/journal.pcbi.1003238 (PMC3784495; doi:10.1371/journal.pcbi.1003238)
Supplement: Figure S8 — Effect of sampling interval and perturbation frequency on the frequency response of residues in TMD1. (A) Power spectral density of all residues except α7 sampled at 0.5, 1, 2 and 5 ps intervals. Vertical green dotted lines intersecting the frequency axis at ∼6 ns−1 represent the upper frequency limit of power spectrum which is assumed to obey distribution. (B) Percent of perturbed Cα atoms identified using different number of TMD simulation cycles ranging from one to 16. (PDF) [file pcbi.1003238.s008.pdf]

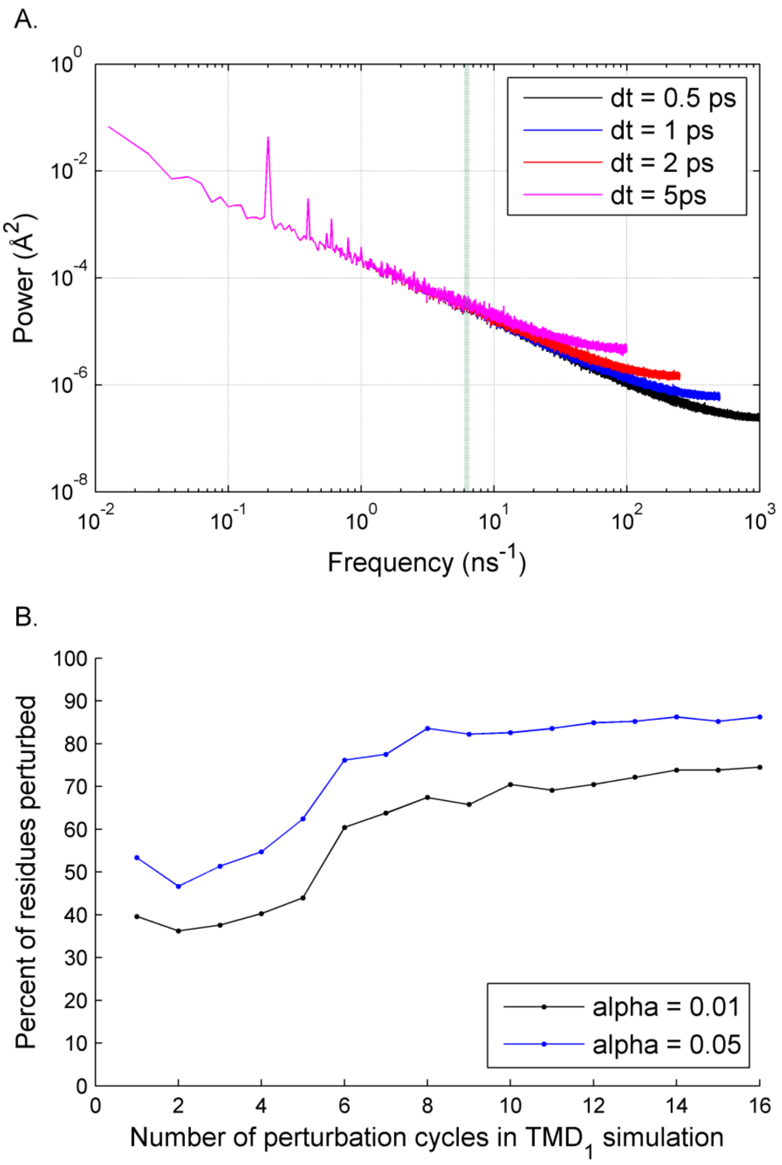

Figure S8. Effect of sampling interval and perturbation frequency on the frequency response of residues in TMD$_1$. (A) Power spectral density of all residues except α7 sampled at 0.5, 1, 2 and 5 ps intervals. Vertical green dotted lines intersecting the frequency axis at ~6 ns^{-1} represent the upper frequency limit of power spectrum which is assumed to obey $1/f^n$ distribution. (B) Percent of perturbed C_α atoms identified using different number of TMD simulation cycles ranging from one to 16.
