# Supplementary material for: Frequency Response of a Protein to Local Conformational Perturbations
Source: PLoS Comput Biol. 2013 Sep 26;9(9):e1003238. doi: 10.1371/journal.pcbi.1003238 (PMC3784495; doi:10.1371/journal.pcbi.1003238)
Supplement: Figure S9 — Power spectral density of various perturbed side-chain dihedral angles in the vicinity of WPD loop. Power component at 0.2 and 0.4 ns−1 are denoted by a square and a triangle, respectively. (PDF) [file pcbi.1003238.s009.pdf]

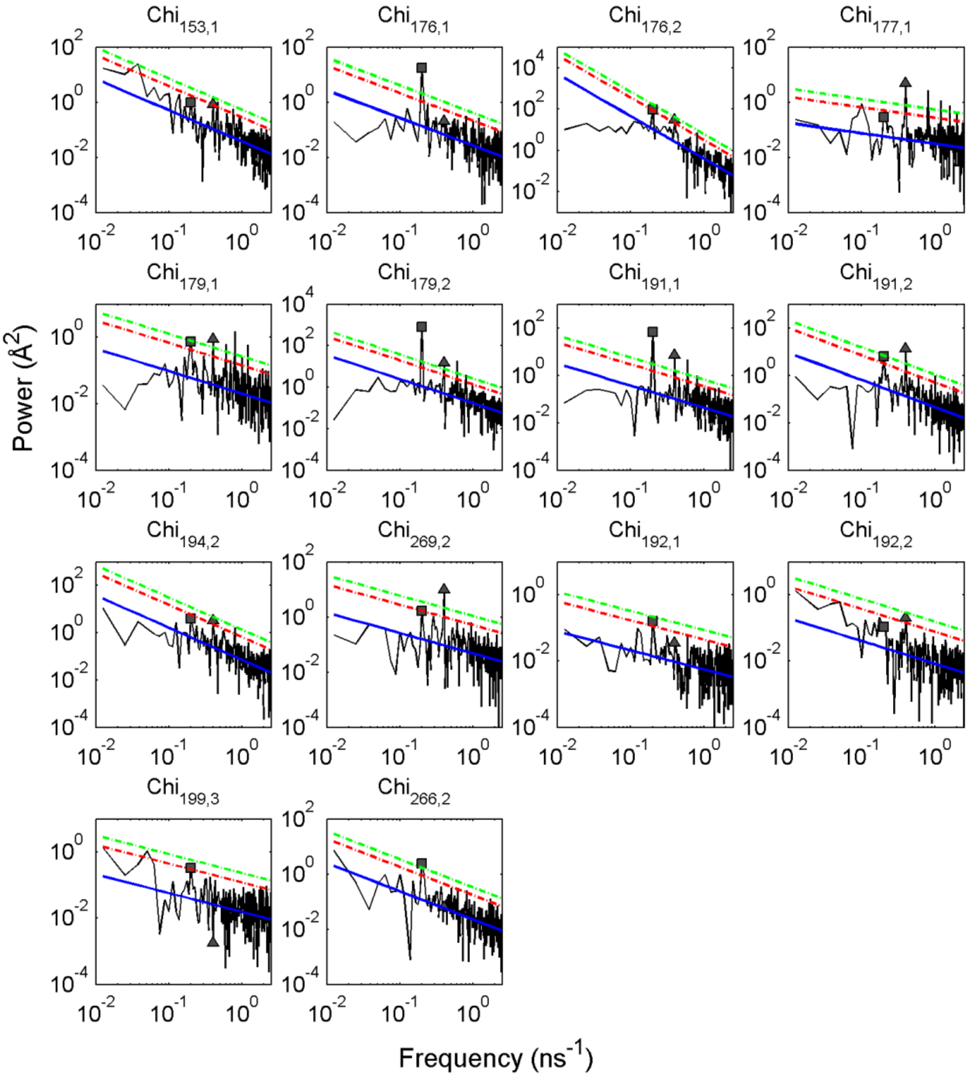

Figure S9. Power spectral density of various perturbed side-chain dihedral angles in the vicinity of WPD loop. Power component at 0.2 and 0.4 ns^{-1} are denoted by a square and a triangle, respectively.
