# Supplementary material for: Frequency Response of a Protein to Local Conformational Perturbations
Source: PLoS Comput Biol. 2013 Sep 26;9(9):e1003238. doi: 10.1371/journal.pcbi.1003238 (PMC3784495; doi:10.1371/journal.pcbi.1003238)
Supplement: Figure S10 — Sensitivity of phase angles of interatomic distances to amplitudes of atomic fluctuations. (A) Two approximately in-phase signals (atomic positions) with a phase difference of π/33 and a unit difference between their amplitudes, and (B) difference (interatomic distance) between the amplitudes of these two signals; new signal is also approximately in-phase with the first two signals. (C) Two approximately in-phase signals with a phase difference of π/33 and 5% difference between their amplitudes, and (D) difference between the amplitudes of these two signals; phase difference between new signal and the first two signals is approximately π/2. (PDF) [file pcbi.1003238.s010.pdf]

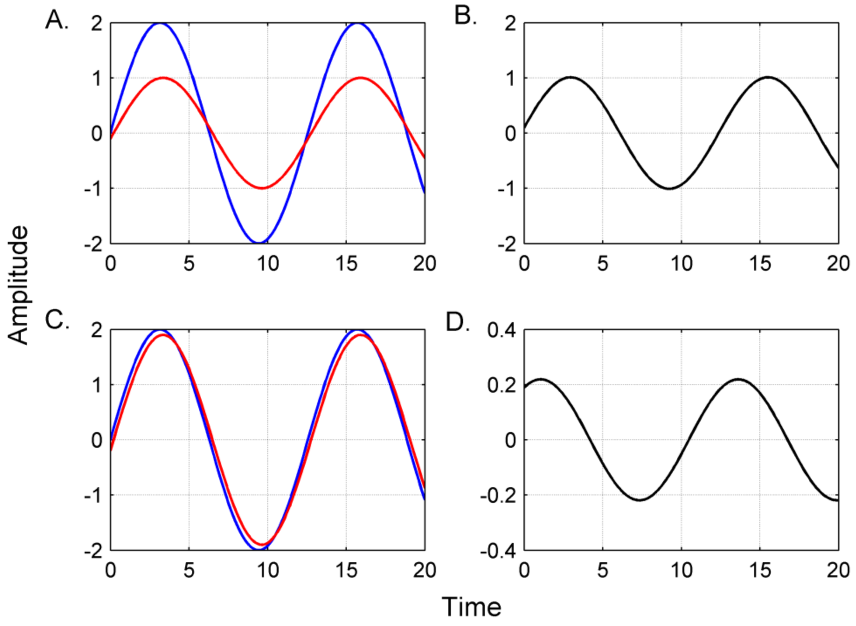

Figure S10. Sensitivity of phase angles of interatomic distances to amplitudes of atomic fluctuations. (A) Two approximately in-phase signals (atomic positions) with a phase difference of $\pi/33$ and a unit difference between their amplitudes, and (B) difference (interatomic distance) between the amplitudes of these two signals; new signal is also approximately in-phase with the first two signals. (C) Two approximately in-phase signals with a phase difference of $\pi/33$ and 5% difference between their amplitudes, and (D) difference between the amplitudes of these two signals; phase difference between new signal and the first two signals is approximately $\pi/2$.
