# Supplementary material for: Frequency Response of a Protein to Local Conformational Perturbations
Source: PLoS Comput Biol. 2013 Sep 26;9(9):e1003238. doi: 10.1371/journal.pcbi.1003238 (PMC3784495; doi:10.1371/journal.pcbi.1003238)
Supplement: Figure S11 — Atomic representations of in-phase fluctuating residues in the third and fourth clusters of atomic variables. (A) The third cluster of in-phase fluctuations consists of coupled motions of the backbone of R-loop, side-chains of Asp181, Phe182 on the WPD loop, and Trp96, Tyr124 and Leu160, which make hydrophobic interactions. (B) The fourth cluster of in-phase fluctuations consists of coupled motions of the backbones of β9, β10 and β11 and side-chains of Asn44, Gln61, Trp100 and Leu160. Out of these four residues, the last two make hydrophobic contacts. In both figures, residues with perturbed side-chains are colored with respect their atoms (nitrogen in blue, oxygen in red, carbon in gray), regions of backbones with perturbed dihedral angles are colored in purple, and perturbed H-bonds are shown with black dashed lines. (PDF) [file pcbi.1003238.s011.pdf]

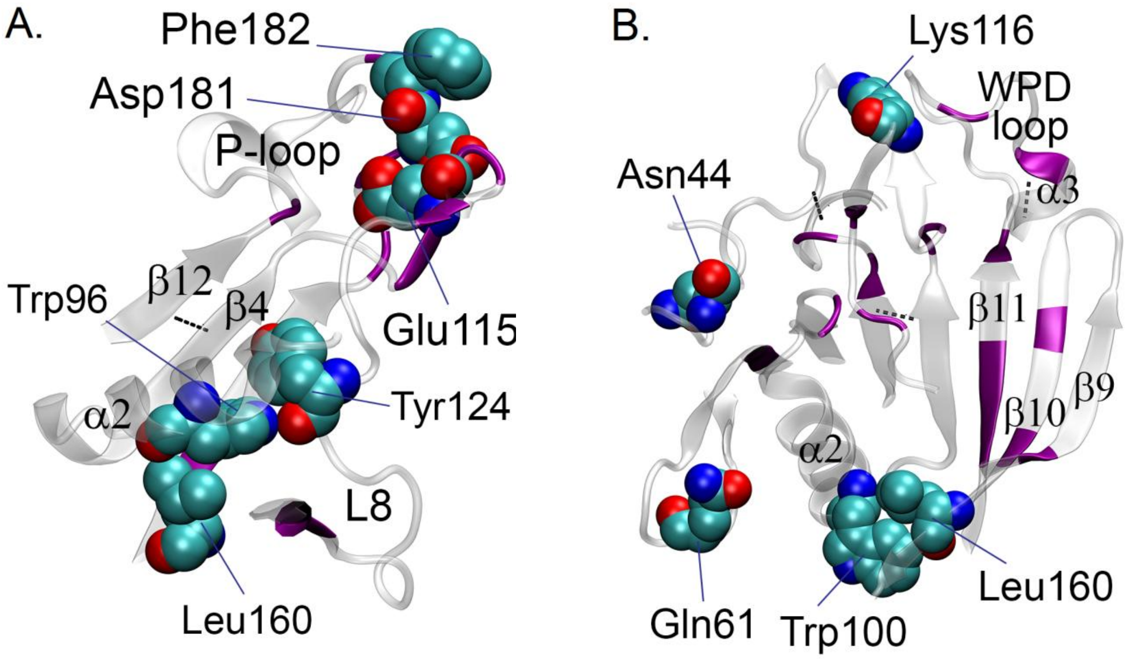

Figure S11. Atomic representations of in-phase fluctuating residues in the third and fourth clusters of atomic variables. (A) The third cluster of in-phase fluctuations consists of coupled motions of the backbone of R-loop, side-chains of Asp181, Phe182 on the WPD loop, and Trp96, Tyr124 and Leu160, which make hydrophobic interactions. (B) The fourth cluster of in-phase fluctuations consists of coupled motions of the backbones of β9, β10 and β11 and side-chains of Asn44, Gln61, Trp100 and Leu160. Out of these four residues, the last two make hydrophobic contacts. In both figures, residues with perturbed side-chains are colored with respect their atoms (nitrogen in blue, oxygen in red, carbon in gray), regions of backbones with perturbed dihedral angles are colored in purple, and perturbed H-bonds are shown with black dashed lines.
