# Supplementary material for: Frequency Response of a Protein to Local Conformational Perturbations
Source: PLoS Comput Biol. 2013 Sep 26;9(9):e1003238. doi: 10.1371/journal.pcbi.1003238 (PMC3784495; doi:10.1371/journal.pcbi.1003238)
Supplement: Figure S12 — Classification of the residues with respect to their frequency responses. Blue and red colored residues have monotonic decreasing and monotonic increasing frequency responses, respectively. Concave functions may be fitted to the frequency responses of yellow residues, indicating an underdamped behavior. No definite trends in Bode plots of white residues have been observed (see Table S5). Transparent regions represent Cα atoms not being perturbed by the TMD potential. (PDF) [file pcbi.1003238.s012.pdf]

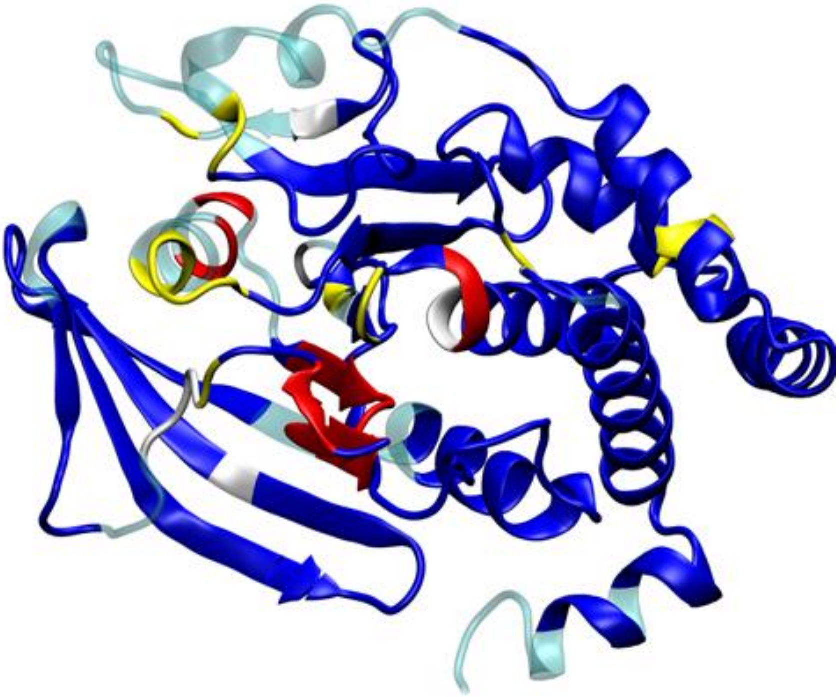

Figure S12. Classification of the residues with respect to their frequency responses. Blue and red colored residues have monotonic decreasing and monotonic increasing frequency responses, respectively. Concave functions may be fitted to the frequency responses of yellow residues, indicating an underdamped behavior. No definite trends in Bode plots of white residues have been observed (see Table S5). Transparent regions represent C_α atoms not being perturbed by the TMD potential.
