# Supplementary material for: Frequency Response of a Protein to Local Conformational Perturbations
Source: PLoS Comput Biol. 2013 Sep 26;9(9):e1003238. doi: 10.1371/journal.pcbi.1003238 (PMC3784495; doi:10.1371/journal.pcbi.1003238)
Supplement: Figure S13 — Trajectory of φ 182 backbone dihedral angle in TMD1 simulation. Black and maroon dashed lines represent the angles adopted by φ 182 in WPDopen and WPDclosed crystal structures. Yellow lines represent the trajectory of φ 182 from WPDopen to WPDclosed conformations, while blue lines represent the trajectory of φ 182 from WPDclosed to WPDopen conformations. (PDF) [file pcbi.1003238.s013.pdf]

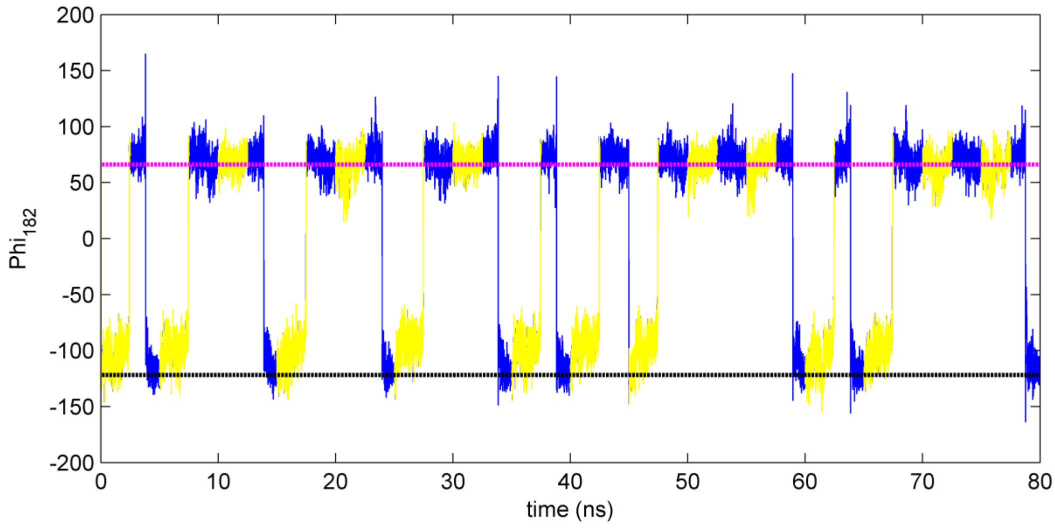

Figure S13. Trajectory of ϕ_{182} backbone dihedral angle in TMD$_1$ simulation. Black and maroon dashed lines represent the angles adopted by ϕ_{182} in WPD$_{open}$ and WPD$_{closed}$ crystal structures. Yellow lines represent the trajectory of ϕ_{182} from WPD$_{open}$ to WPD$_{closed}$ conformations, while blue lines represent the trajectory of ϕ_{182} from WPD$_{closed}$ to WPD$_{open}$ conformations.
