# Supplementary material for: Frequency Response of a Protein to Local Conformational Perturbations
Source: PLoS Comput Biol. 2013 Sep 26;9(9):e1003238. doi: 10.1371/journal.pcbi.1003238 (PMC3784495; doi:10.1371/journal.pcbi.1003238)
Supplement: Figure S14 — WPD loop conformational transition on the reduced PC planes. Small black dots represent the whole trajectory of WPD loop during TMD1 simulation. Pink and green lines represent the WPD loop trajectory during the first loop closing (WPDopen→WPDclosed) and the first loop opening (WPDclosed→WPDopen), respectively. Blue and red filled circles represent the WPDopen and WPDclosed crystal structures, respectively. Yellow filled square and purple filled circles connected by gray arrows denote the trajectory of WPD loop in the absence of active water molecules in the WPDclosed state. Light gray crosses represent an equilibrium simulation in WPDclosed conformation. The numbers in parenthesis on the axis labels denote the percentage of explanation offered by PCA. (PDF) [file pcbi.1003238.s014.pdf]

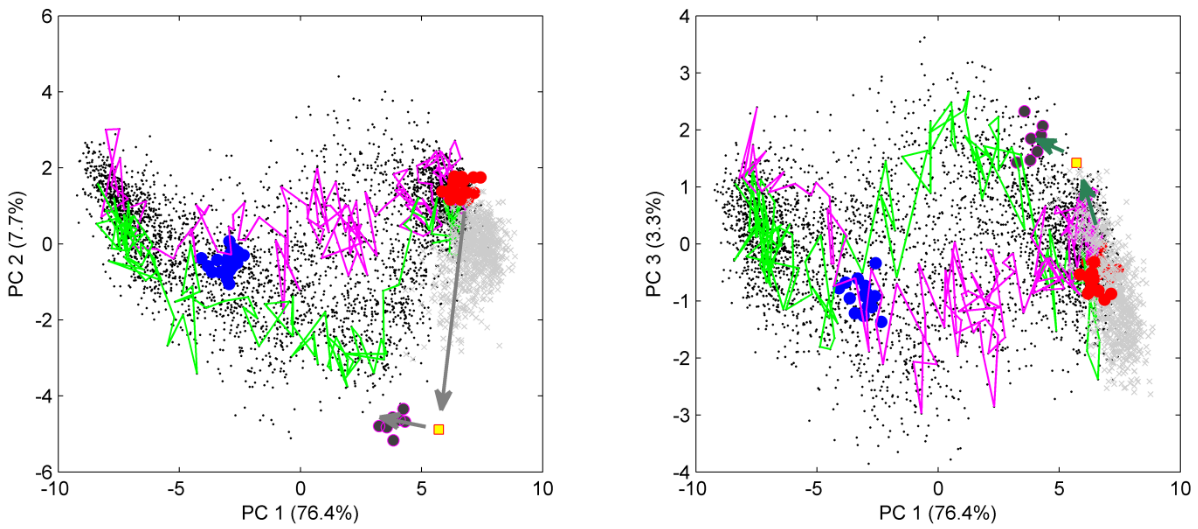

Figure S14. WPD loop conformational transition on the reduced PC planes. Small black dots represent the whole trajectory of WPD loop during TMD_1 simulation. Pink and green lines represent the WPD loop trajectory during the first loop closing ($WPD_{open} \rightarrow WPD_{closed}$) and the first loop opening ($WPD_{closed} \rightarrow WPD_{open}$), respectively. Blue and red filled circles represent the WPD_{open} and WPD_{closed} crystal structures, respectively. Yellow filled square and purple filled circles connected by gray arrows denote the trajectory of WPD loop in the absence of active water molecules in the WPD_{closed} state. Light gray crosses represent an equilibrium simulation in WPD_{closed} conformation. The numbers in parenthesis on the axis labels denote the percentage of explanation offered by PCA.
