# Supplementary material for: Frequency Response of a Protein to Local Conformational Perturbations
Source: PLoS Comput Biol. 2013 Sep 26;9(9):e1003238. doi: 10.1371/journal.pcbi.1003238 (PMC3784495; doi:10.1371/journal.pcbi.1003238)
Supplement: Figure S15 — Histograms of the backbone dihedral angles of WPD loop residues. Blue and red lines represent the averages of the dihedral angles adopted during the first 50 ps and the last 50 ps in the first WPD loop transition. (PDF) [file pcbi.1003238.s015.pdf]

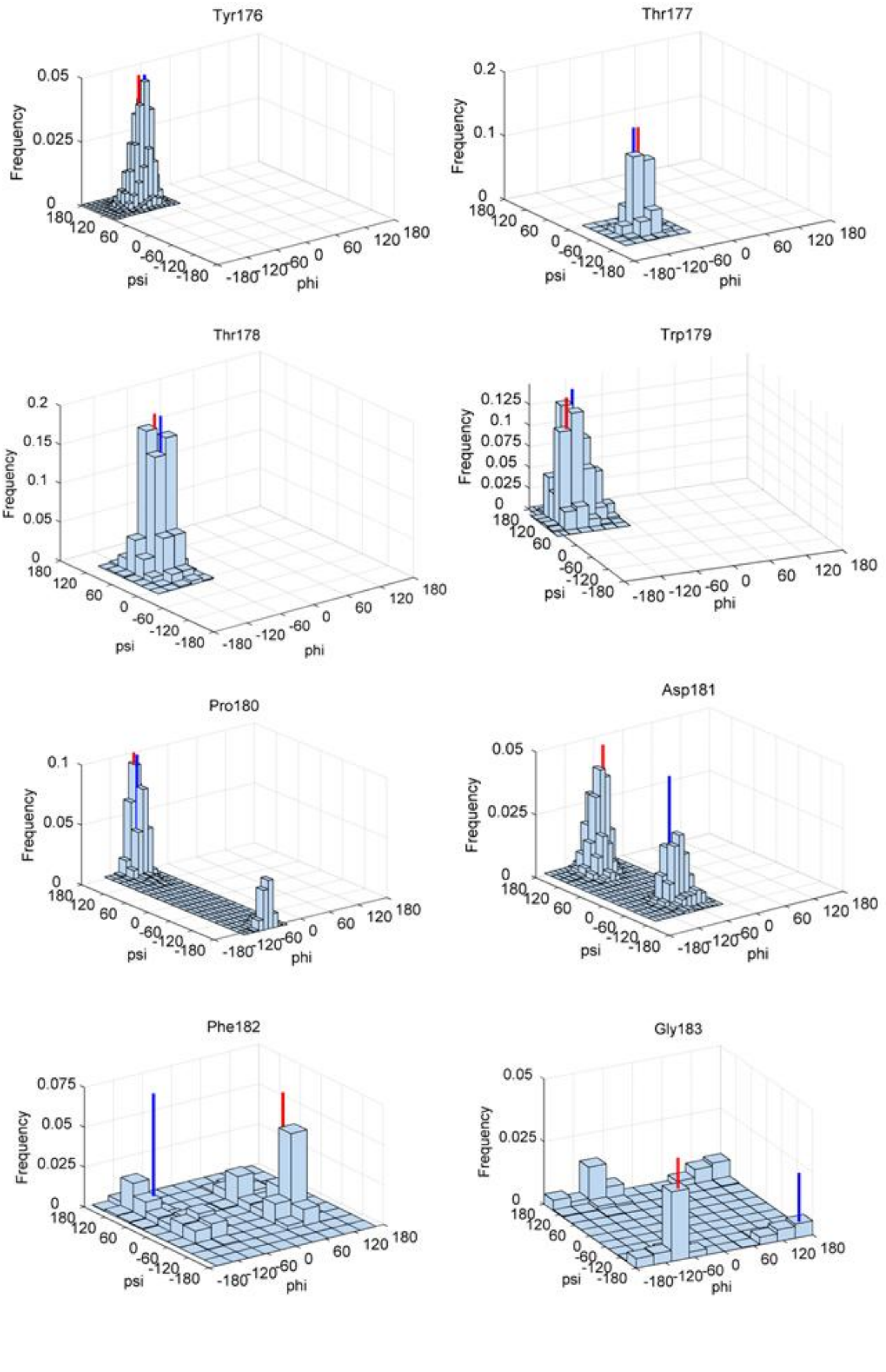
Tyr176
Thr177
Thr178
Trp179
Pro180
Asp181
Phe182
Gly183
Frequency
psi
phi

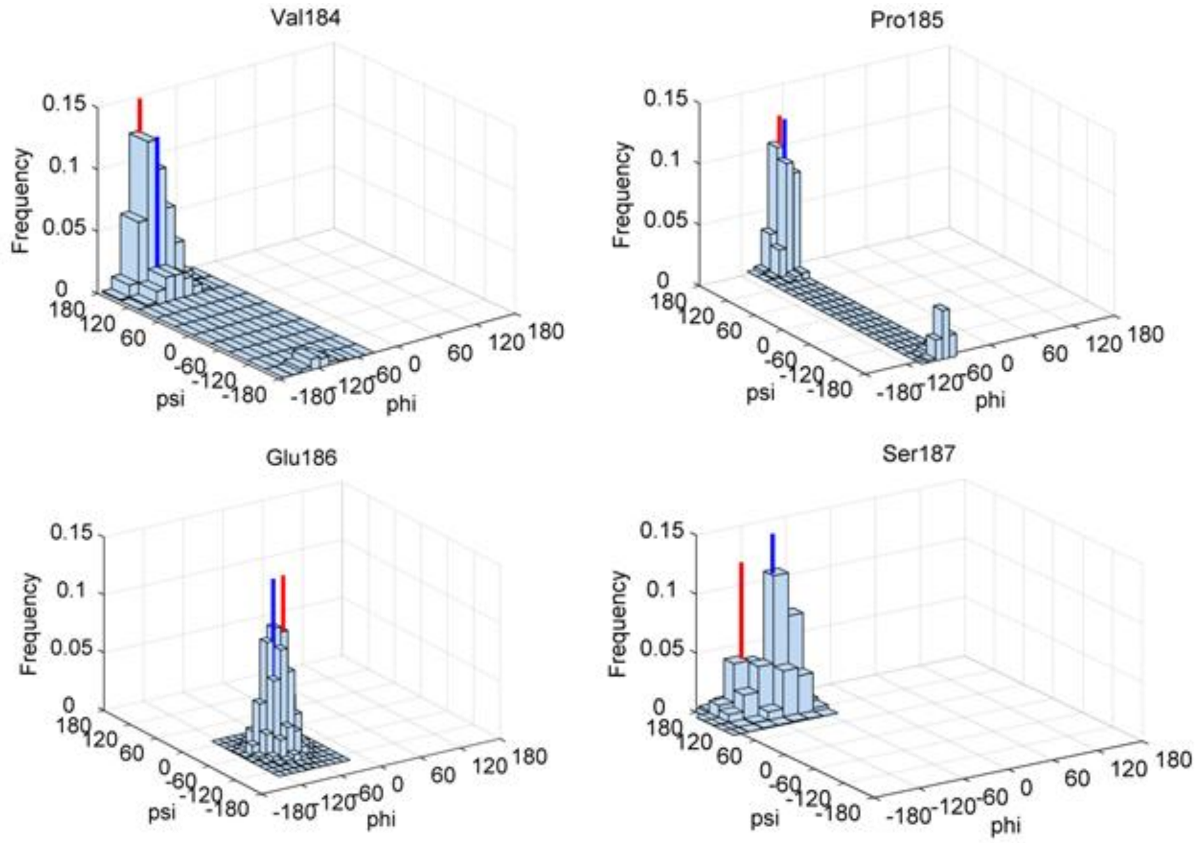

Figure S15. **Histograms of the backbone dihedral angles of WPD loop residues.** Blue and red lines represent the averages of the dihedral angles adopted during the first 50 ps and the last 50 ps in the first WPD loop transition.
