# Supplementary material for: Frequency Response of a Protein to Local Conformational Perturbations
Source: PLoS Comput Biol. 2013 Sep 26;9(9):e1003238. doi: 10.1371/journal.pcbi.1003238 (PMC3784495; doi:10.1371/journal.pcbi.1003238)
Supplement: Figure S16 — Structural analysis of WPD loop in TMD′ simulation. (A) RMSD of the WPD loop from its conformation in WPDopen (dark blue) and WPDclosed (light blue) crystal structures. (B) Trajectory of φ 182 dihedral angle. Lines and coloring are identical to those in Figure S13. (C) Histogram of the backbone dihedral angles of Phe182. (PDF) [file pcbi.1003238.s016.pdf]

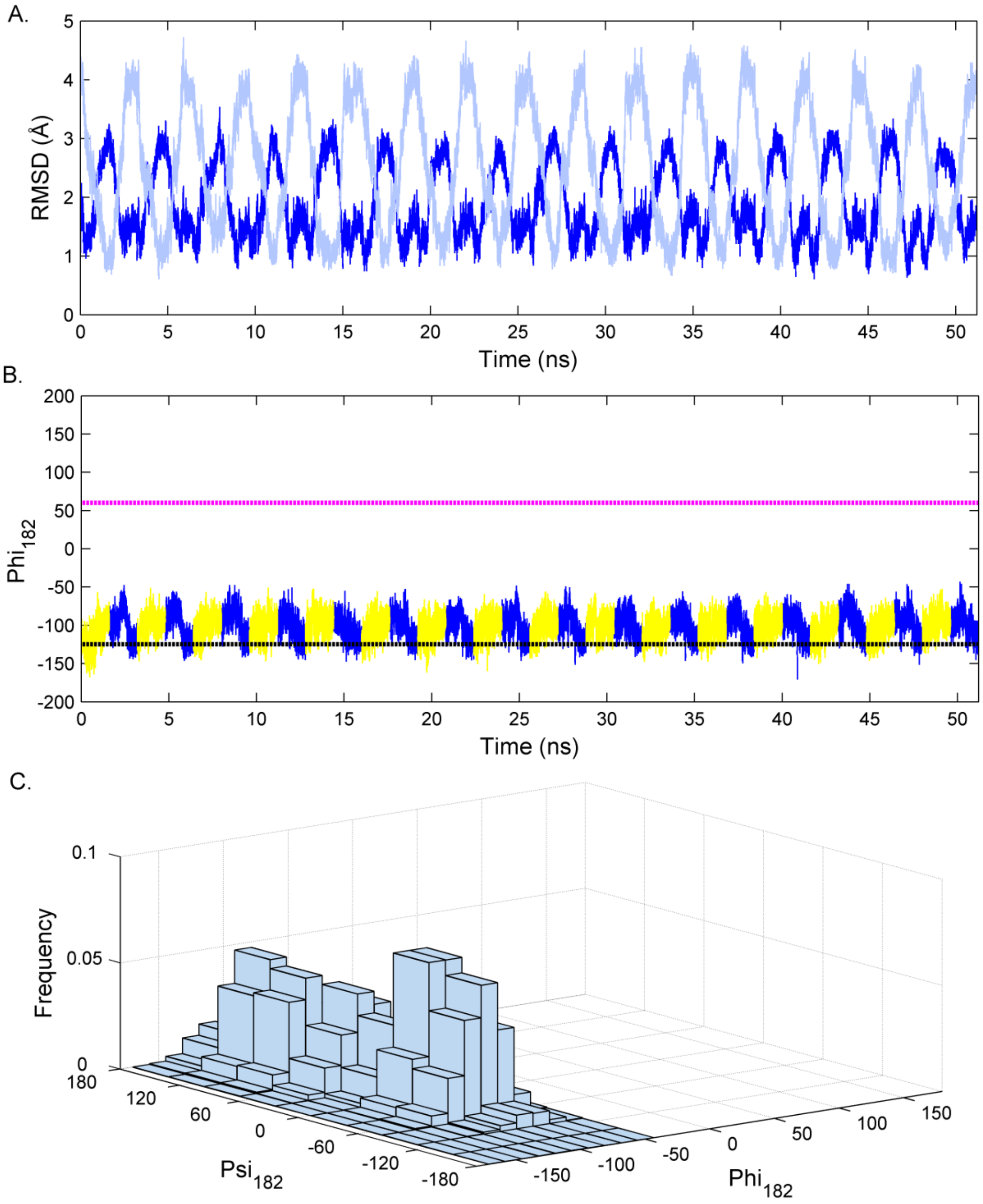

Figure S16. Structural analysis of WPD loop in TMD′ simulation. (A) RMSD of the WPD loop from its conformation in WPD_{open} (dark blue) and WPD_{closed} (light blue) crystal structures. (B) Trajectory of ϕ_{182} dihedral angle. Lines and coloring are identical to those in Figure S13. (C) Histogram of the backbone dihedral angles of Phe182.
