# Supplementary material for: Frequency Response of a Protein to Local Conformational Perturbations
Source: PLoS Comput Biol. 2013 Sep 26;9(9):e1003238. doi: 10.1371/journal.pcbi.1003238 (PMC3784495; doi:10.1371/journal.pcbi.1003238)
Supplement: Figure S17 — Comparison of WPD loop conformational transitions in TMD simulations with different spring constants on the reduced PC planes. Projection of WPD loop transitions on (A) PC1–PC2, and (B) PC1–PC3 planes. Black and blue circles represent TMD simulations with spring constant equal to 3000 kcal·mol−1·Å−2 and 500 kcal·mol−1·Å−2, respectively. (C) Transition of WPD loop in the first cycle of both simulations on PC1–PC2 plane. Black (TMD1) and blue lines (TMD′) represent the WPD loop trajectory during the first loop closing, while red (TMD1) and green (TMD′) represent the first loop opening. (PDF) [file pcbi.1003238.s017.pdf]

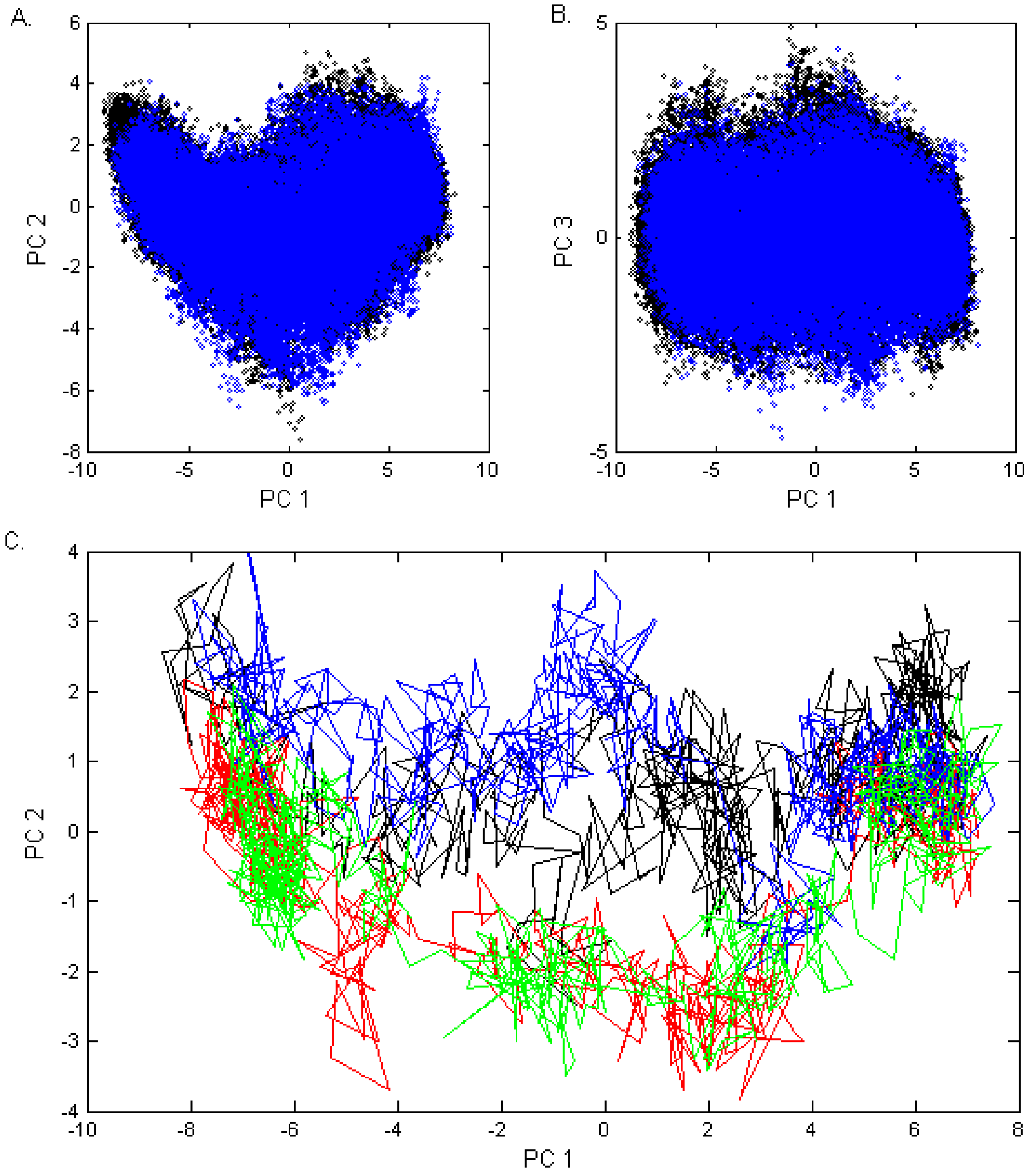

Figure S17. Comparison of WPD loop conformational transitions in TMD simulations with different spring constants on the reduced PC planes. Projection of WPD loop transitions on (A) PC1-PC2, and (B) PC1-PC3 planes. Black and blue circles represent TMD simulations with spring constant equal to 3000 kcal·mol^{-1}·Å^{-2} and 500 kcal·mol^{-1}·Å^{-2}, respectively. (C) Transition of WPD loop in the first cycle of both simulations on PC1-PC2 plane. Black (TMD_1) and blue lines (TMD′) represent the WPD loop trajectory during the first loop closing, while red (TMD_1) and green (TMD′) represent the first loop opening.
