# Supplementary material for: Frequency Response of a Protein to Local Conformational Perturbations
Source: PLoS Comput Biol. 2013 Sep 26;9(9):e1003238. doi: 10.1371/journal.pcbi.1003238 (PMC3784495; doi:10.1371/journal.pcbi.1003238)
Supplement: Figure S18 — Time and frequency domain responses of first order lag and lead-lag systems. (A) Step, and (B) frequency responses of a first order lag system. In (B), breakpoint frequency is equal to 1/τp = 0.1 rad/s. Unit step responses of lead-lag processes with (C) τz/τp<1, and (D) τz/τp>1. Magnitude Bode plots of lead-lag processes with (E) τz/τp<1, and (F) τz/τp>1. (PDF) [file pcbi.1003238.s018.pdf]

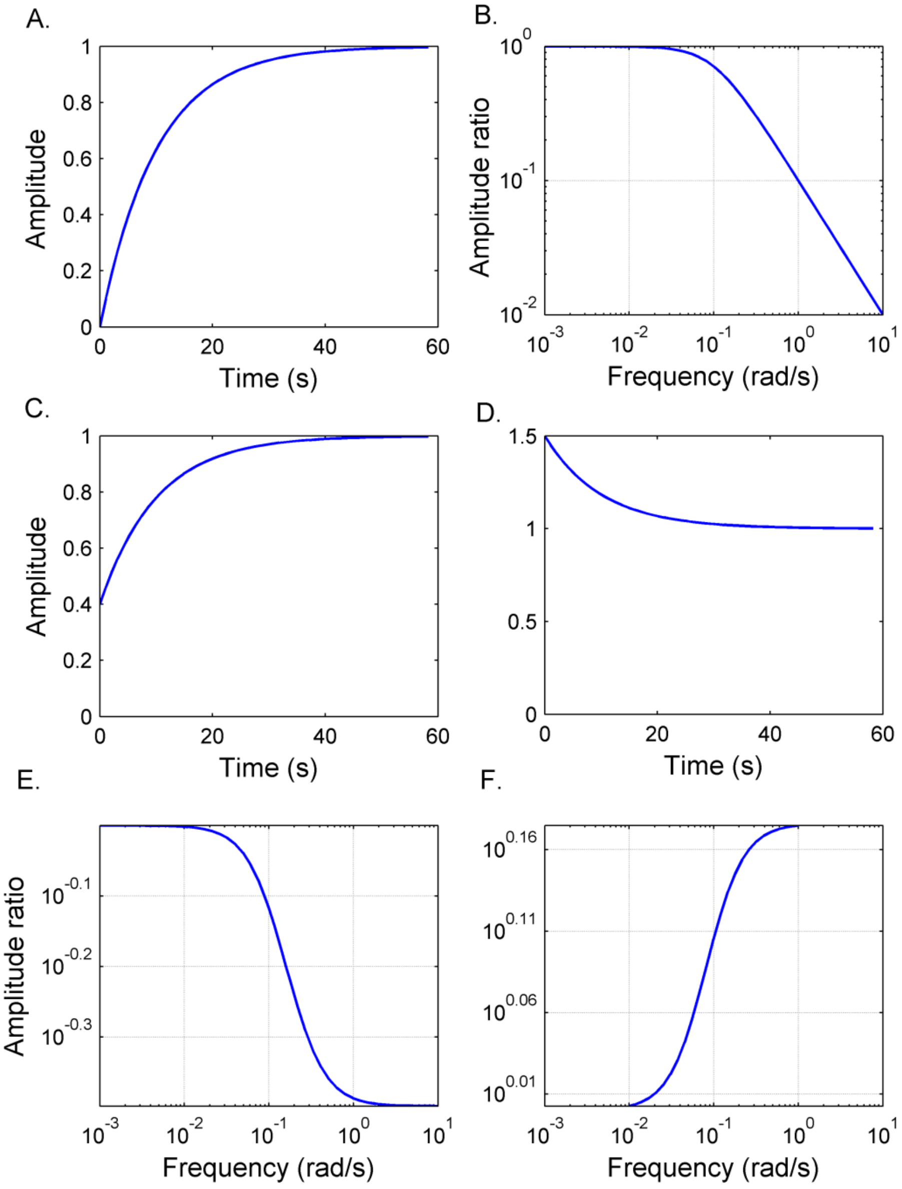

Figure S18. Time and frequency domain responses of first order lag and lead-lag systems. (A) Step, and (B) frequency responses of a first order lag system. In (B), breakpoint frequency is equal to $1/\tau_p = 0.1$ rad/s. Unit step responses of lead-lag processes with (C) $\tau_z/\tau_p < 1$, and (D) $\tau_z/\tau_p > 1$. Magnitude Bode plots of lead-lag processes with (E) $\tau_z/\tau_p < 1$, and (F) $\tau_z/\tau_p > 1$.
