# Supplementary material for: Frequency Response of a Protein to Local Conformational Perturbations
Source: PLoS Comput Biol. 2013 Sep 26;9(9):e1003238. doi: 10.1371/journal.pcbi.1003238 (PMC3784495; doi:10.1371/journal.pcbi.1003238)
Supplement: Table S1 — PDB IDs of the crystal structures used in the current study. (PDF) [file pcbi.1003238.s019.pdf]

Table S1. PDB IDs of the crystal structures used in the current study.

WPD_{open} and $L16_{I}$ conformation[(a)]	WPD_{open} and $L16_{II}$ conformation[(b)]	WPD_{closed} and $L16_{I}$ conformation[(c)]	WPD_{closed} and $L16_{II}$ conformation[(d)]
1BZH, 1NNY, 1NO6, 1NWL, 1NZ7, 1ONY, 1ONZ, 1PH0, 1JF7, 1NL9, 1OEO, 1OET, 1OEU, 1OEV, 1PYN, 1T48, 1T49, 1T4J, 2CM2, 2F6F	1G7F, 2HNP, 2HNQ	1EEN, 1G1F, 1G1G, 1G1H, 1G7G, 1KAK, 1KAV, 1PXH, 2CMA, 2VEU, 2VEV, 2CM7, 2VEW, 2VEX, 2VEY	1AAX, 1BZC, 1C83, 1C84, 1C85, 1C86, 1C87, 1ECV, 1GFY, 1L8G, 1PTT, 1PTU, 1PTY, 1Q1M, 1SUG, 1WAX, 1XBO, 2AZR, 2F6T, 2F71, 2NTA

[(a)] WPD loop adopts open conformation and L16 adopts $L16_{I}$ conformation.
[(b)] WPD loop adopts open conformation and L16 adopts $L16_{II}$ conformation.
[(c)] WPD loop adopts closed conformation and L16 adopts $L16_{I}$ conformation.
[(d)] WPD loop adopts closed conformation and L16 adopts $L16_{II}$ conformation.
