# Supplementary material for: Frequency Response of a Protein to Local Conformational Perturbations
Source: PLoS Comput Biol. 2013 Sep 26;9(9):e1003238. doi: 10.1371/journal.pcbi.1003238 (PMC3784495; doi:10.1371/journal.pcbi.1003238)
Supplement: Table S2 — Overlap of residue displacements of reconstructed trajectories determined from low frequency TMD simulations. (PDF) [file pcbi.1003238.s020.pdf]

Table S2. Overlap of residue displacements of reconstructed trajectories determined from low frequency TMD simulations.

Overlap of the first eigenvectors	Residues 2 to 278	R and WPD loops excluded
P_{TMD1}[(a)] and P_{TMD2}	0.98	0.91
P_{TMD1} and P_{TMD3}	0.98	0.92
P_{TMD2} and P_{TMD3}	0.98	0.92

[(a)] List of TMD simulations is given in Table S4. P_{TMD1}, for instance, denotes the first eigenvector determined from the reconstructed trajectories in TMD_1.
