# Supplementary material for: Frequency Response of a Protein to Local Conformational Perturbations
Source: PLoS Comput Biol. 2013 Sep 26;9(9):e1003238. doi: 10.1371/journal.pcbi.1003238 (PMC3784495; doi:10.1371/journal.pcbi.1003238)
Supplement: Table S3 — Number of perturbed Cα atoms determined from low frequency TMD simulations. (PDF) [file pcbi.1003238.s021.pdf]

Table S3. Number of perturbed C_α atoms determined from low frequency TMD simulations.

TMD simulations	TMD_1	TMD_2	TMD_3
TMD_1	[(a)]257/222	[(b)]233/200	238/197
TMD_2		248/211	229/196
TMD_3			256/221

[(a)] Diagonal elements are the numbers of C_α atoms perturbed at $\alpha = 0.05$ and $\alpha = 0.01$ (separated by slash), respectively, in the simulation represented by the corresponding row or column.

[(b)] Off-diagonal elements are the numbers of common C_α atoms perturbed at $\alpha = 0.05$ and $\alpha = 0.01$ (separated by slash), respectively, in both simulations represented by the corresponding row and column.
