# Supplementary material for: Frequency Response of a Protein to Local Conformational Perturbations
Source: PLoS Comput Biol. 2013 Sep 26;9(9):e1003238. doi: 10.1371/journal.pcbi.1003238 (PMC3784495; doi:10.1371/journal.pcbi.1003238)
Supplement: Table S4 — List of TMD simulations performed and analyzed in the current study. (PDF) [file pcbi.1003238.s022.pdf]

Table S4. List of TMD simulations performed and analyzed in the current study.

TMD simulations	Cycling period (ps)	Frequency (f_o) (ns^{-1})	Frequency[a] (w_o) (rad/ns)	Total simulation period[b] (ns)
TMD_1	5000	0.200	1.26	80.0
TMD_2	2000	0.500	3.14	32.0
TMD_3	1200	0.833	5.23	19.2
TMD_4	800	1.25	7.9	12.8
TMD_5	500	2.00	12.6	8.00
TMD_6	350	2.86	17.9	5.60
TMD_7	200	5.00	31.4	3.20
TMD_8	150	6.67	41.9	2.40
TMD_9	100	10.0	62.8	1.60
TMD_{10}	70	14.3	89.7	1.12
TMD_{11}	50	20.0	126	0.80
TMD_{12}	40	25.0	157	0.64
TMD_{13}	30	33.3	209	0.48

[a] Power spectral density and Bode plots are plotted with respect to frequencies represented in $ns^{-1}$and rad/ns, respectively.

[b] Total simulation period is 16×cyling period for each simulation, corresponding to 16 cycles of WPD loop opening and closing motion.
