# Supplementary material for: Frequency Response of a Protein to Local Conformational Perturbations
Source: PLoS Comput Biol. 2013 Sep 26;9(9):e1003238. doi: 10.1371/journal.pcbi.1003238 (PMC3784495; doi:10.1371/journal.pcbi.1003238)
Supplement: Table S5 — Classification of magnitude Bode plot data of Cα atoms. (PDF) [file pcbi.1003238.s023.pdf]

Table S5. Classification of magnitude Bode plot data of C_α atoms.

Type of function to which frequency response data may be fitted	Percentage of residues modeled with lead-lag transfer functions	Percentage of residues not modeled with lead-lag transfer functions
Monotonic decreasing	55	25
Monotonic increasing	5.0	3.3
No trend	-	7.8
Concave	-	3.9
