# Supplementary material for: Frequency Response of a Protein to Local Conformational Perturbations
Source: PLoS Comput Biol. 2013 Sep 26;9(9):e1003238. doi: 10.1371/journal.pcbi.1003238 (PMC3784495; doi:10.1371/journal.pcbi.1003238)
Supplement: Text S1 — Single eigenvalue representation of collective residue displacements. (PDF) [file pcbi.1003238.s024.pdf]

Text S1. Single eigenvalue representation of collective residue displacements

A single C_α displacement vector representing the collective conformational change of PTP1B is required to make a comparison with the experimental residue displacements. Projection of the raw trajectories on the base frequency, however, yields filtered atomic trajectories at different phases, e.g. phase differences up to $\pi/10$ (~250 ps) were seen between the reconstructed trajectories of residues Thr178 to Ser187 (Figure S4A). Here, a simple method is suggested to determine collective residue displacements for comparison with crystal structures. Reconstructed trajectory matrix of C_α atoms is made up weighted sum of sine and cosine components at the same frequency, hence its covariance matrix can be decomposed into two non-zero eigenvalues. If the first eigenvalue is sufficiently larger than the second one, projection of the reconstructed trajectory on the first eigenvector, named reconstructed in-phase trajectory, can represent the displacement of a single residue, or a set of residues during the conformational transition of PTP1B. The first eigenvalues obtained by employing PCA on the reconstructed trajectories of Thr178 to Ser187 and Glu2 to Ala278 explained 99% (Figure S4B) and 93% (Figure S4C) of the fluctuations of the examined regions, respectively. These significantly high eigenvalues justify the representation of collective atomic displacements with a single eigenvector.
