# Supplementary material for: Frequency Response of a Protein to Local Conformational Perturbations
Source: PLoS Comput Biol. 2013 Sep 26;9(9):e1003238. doi: 10.1371/journal.pcbi.1003238 (PMC3784495; doi:10.1371/journal.pcbi.1003238)
Supplement: Text S2 — Conformations of L16 adopted in the crystal structures of PTP1B. (PDF) [file pcbi.1003238.s025.pdf]

Text S2. Conformations of L16 adopted in the crystal structures of PTP1B

Average conformations adopted in the WPD_{open} and WPD_{closed} crystal structures (Table S1) were determined, and amplitude of C_{α} atomic displacements (*D*) between these two structures computed. Correlation of the experimental and predicted amplitudes of displacements of residues 2 to 278 was found to be 0.91. A more reliable metric may be obtained when R and WPD loop regions are excluded from this comparison, since TMD potential was directly applied on these regions. Excluding these two regions, correlation of residue displacement profiles was found to be 0.57. Figure S5 showed L16 was the only region showing a significant discrepancy between experimental and predicted residue displacements. Visualization of the crystal structures shows (Figure S6) that L16, at a distance of ~30-40 Å from Asp181, may adopt two different conformations. In 15 crystal structures of WPD_{closed} conformation, L16 adopts a conformation ($L16_{I}$) similar to that seen in 20 of the WPD_{open} crystal structures, with a RMSD of 0.49 Å between the conformation averages of each group. In 21 crystal structures of WPD_{closed} conformation and three structures of WPD_{open} conformation, on the other hand, L16 adopts an alternative conformation ($L16_{II}$), with a RMSD of 2.4 Å from the average conformation of L16 in WPD_{open} crystal structures. This shows that the conformational transitions seen in L16 and WPD loop are not necessarily coupled, and L16 may adopt different conformations in WDP_{closed} and, to a lesser extent, in WPD_{open} crystal structures. The initial WPD_{open} crystal structure in all TMD simulations adopted $L16_{I}$ conformation, and L16 did not make a transition to $L16_{II}$ conformation in any of the TMD simulations. Hence, the total set of experimental structures was reduced to a set of crystal structures only in $L16_{I}$ conformation to be used in comparison with the current method predictions, and a higher consistency between computations and experiments were obtained (see Figure 1D and Table 1).
