# Supplementary material for: Frequency Response of a Protein to Local Conformational Perturbations
Source: PLoS Comput Biol. 2013 Sep 26;9(9):e1003238. doi: 10.1371/journal.pcbi.1003238 (PMC3784495; doi:10.1371/journal.pcbi.1003238)
Supplement: Text S3 — Determination of a single reconstructed trajectory for each Cα atom. (PDF) [file pcbi.1003238.s026.pdf]

Text S3. Determination of a single reconstructed trajectory for each C_α atom

Fluctuations of C_α atoms on the base frequency have to be treated separately for each C_α atom to obtain single filtered trajectories. The first eigenvector obtained by employing PCA on the three reconstructed Cartesian coordinates of a C_α atom gives the principal (dominant) motion of that C_α atom. If the first eigenvalue is sufficiently larger than the other two, then it may be assumed that the first eigenvector can faithfully represent the fluctuations of the C_α atom, on which PCA has been employed. Hence, PCA was employed on the three Cartesian coordinates of each C_α atom of residues between Met1 and Asp298. For 272 C_α atoms, the first eigenvector was found to explain more than 75% of the fluctuations, justifying that three Cartesian coordinates can be reduced to a single residue coordinate. Since dihedral angles and H-bond distances can each be represented by single variables, such a complication does not arise for these variables.
