# Supplementary material for: Frequency Response of a Protein to Local Conformational Perturbations
Source: PLoS Comput Biol. 2013 Sep 26;9(9):e1003238. doi: 10.1371/journal.pcbi.1003238 (PMC3784495; doi:10.1371/journal.pcbi.1003238)
Supplement: Text S4 — Robustness of the frequency response method with respect of resolution of power spectra. (PDF) [file pcbi.1003238.s027.pdf]

Text S4. Robustness of the frequency response method with respect of resolution of power spectra

In the current frequency analysis, perturbed atomic variables were identified using one-sided hypothesis tests at the base frequency. Examining the power spectral density of atomic variables shows that the first ~500 frequency components (f < ~6 ns^{-1} for TMD_1 simulation) obey $1/f^n$ distribution, while higher frequency components are likely to be affected by aliasing (Figure S8A). Additionally, among the first ~500 frequency components, the first ~10 components are seen to deviate seriously from $\sim 1/f^n$ distribution. Hence, frequency components between ~10 and ~500 were used to determine the one-sided confidence intervals. In the current study, the base frequency corresponds to the 16th frequency component in all simulations since 16 perturbation cycles were performed, and identification of perturbed atomic variables was based on a simulation of 80 ns length. To justify the validity of the current analysis, the following question should be answered. Do the number of perturbation cycles and simulation lengths, i.e. resolution of power spectrum, affect the identification results?

First, we examine how number of perturbation cycles (base frequency component) affects the identification of atomic variables. The analysis above was repeated on a single cycle (75 ns-80 ns period) on TMD_1 and increasing the cycles one by one (e.g. 70 ns-80 ns and 60 ns-80 ns comprised two and four cycles, respectively) 16 cycles of the whole TMD_1 simulation was covered. Percent of perturbed C_α atoms at $\alpha = 0.05$ equilibrates at ~85% after ~8-10 cycles (Figure S8B), confirming the observation that the frequency components above the first ~10 frequency components obey $1/f^n$ distribution. Hence, the number of employed perturbation cycles in the current method is convenient for robust identification of perturbed atomic variables.

Then, we examine whether simulation lengths may affect identification of perturbed atomic variables. To this end, we determined the number of simultaneously perturbed C_{α} atoms at α=0.05 and α=0.01 in different couples of TMD_1, TMD_2 and TMD_3 simulations, with total simulation lengths of 80 ns, 32 ns and 19.2 ns respectively. It is important to note that the base frequencies of these three simulations (0.2 ns^{-1}, 0.5 ns^{-1} and 0.83 ns^{-1}) are also different from each other. Over 90% of all residues (1 to 298) were found to be simultaneously perturbed in couples of TMD simulations (Table S3), confirming the robustness of the current method to simulation length and base frequency. Decreasing the simulation length further, however, increases the perturbation frequency above a certain limit (discussed in the main text) and attenuates the fluctuation of atomic variables. Hence the analysis should be limited to perturbation frequencies lower than ~0.8 ns^{-1}.
