# Supplementary material for: Frequency Response of a Protein to Local Conformational Perturbations
Source: PLoS Comput Biol. 2013 Sep 26;9(9):e1003238. doi: 10.1371/journal.pcbi.1003238 (PMC3784495; doi:10.1371/journal.pcbi.1003238)
Supplement: Text S5 — Are trajectories produced by TMD simulations realistic? (PDF) [file pcbi.1003238.s028.pdf]

Text S5. Are trajectories produced by TMD simulations realistic?

One of the important determinants of WPD loop closure is the rotation of the peptide bond between Asp181 (*i*+1 residue) and Phe182 (*i*+2 residue) from a type I to type II β-turn [90]. In the current study, restrained TMD simulations were conducted using a spring constant *k* equal to 3000 $kcal \cdot mol^{-1} \cdot Å^{-2}$. This value was chosen by trial and error to enable a full opening/closing of the WPD loop; type I to type II β-turn transition was not observed for smaller values of *k*. It should be noted that even with the current value of *k*, the conformational transition of β-turn was realized in nine of 16 cycles (~56%), as monitored by ϕ_{182} backbone dihedral angle (Figure S13).

In order to check the quality of TMD simulations at the current *k* value, i.e. whether the resulting trajectories were realistic, three different analyses were performed. First, WPD loop trajectories were analyzed on reduced principal component (PC) plane (Figure S14). PCA was employed on the WPD loop C_α atomic trajectories obtained from TMD_1 simulation (small black dots), crystal structures in WPD_{open} (blue filled circles) and WPD_{closed} (red filled circles) conformations, an equilibrium MD simulation in WPD_{closed} conformation (light gray crosses) and an MD simulation starting from WPD_{closed} conformation with crystal structure waters removed (yellow square and purple filled circles, connected by gray lines). The last two of these simulations were taken a previous study [52], in which it was found that the absence of active site waters moved WPD loop to an "intermediate" conformation between WPD_{closed} and WPD_{open} conformations and this intermediate conformation was similar to an experimental intermediate structure of WDP loop in PTPRR, which is another member of PTP family. Here on the reduced PC plane, the yellow square represents the conformation of WPD loop when a water molecule (named W_{O6} water) entered the active site, triggering the transition to the intermediate conformation [52], and purple filled circles denote

representative intermediate structures at the end of 20 ns simulation following the entrance of W_{O6} water. These intermediate conformations are not spanned during the equilibrium simulations in WPD_{closed} conformation (light gray circles). Highlighting the first WPD loop opening trajectory (green) representative of the general $WPD_{closed} \rightarrow WPD_{open}$ transition in TMD simulations, it is seen that the trajectory that leads to the intermediate WPD loop conformation and the opening trajectory of WPD loop in TMD simulations are close and parallel on PC1-PC2 plane and overlap on PC1-PC3 plane. Initialization of the MD simulation with the removal of active site waters from the WPD_{closed} structure presumably caused the initial jump to yellow square on the outer boundary of WPD loop opening trajectories on PC1-PC2 plane, but as new water molecules in the active site were equilibrated, intermediate WPD loop conformation tends to converge to the WPD loop opening trajectories in TMD simulations.

Then, histograms of WPD loop backbone dihedral angles were plotted to determine whether these dihedral angles visited energetically unfavorable regions during the whole TMD_1 simulation (Figure S15). Except those of Phe182 and Gly183, distributions of backbone dihedral angles of all residues reside in the traditionally accessible regions. Blue and red lines in the histograms show the initial and final dihedral angles adopted by each residue, and distributions of dihedral angles are accumulated around these regions, showing the absence of intermediate and untestable conformer states. The wide range of angles adopted by ψ_{182} may be initially seen as an artifact of the k value used in the TMD method, but a similar distribution of ψ_{182} angle obtained at a lower k value (see the next paragraph) rejects this view.

Last, we repeated the same analysis performing 16 cycles of TMD simulations (named TMD′) at a k value of 500 kcal·mol^{-1}·Å^{-2}. Period of each cycle was taken to be 3.2 ns, hence frequency of the perturbation force was 0.31 ns^{-1}, much lower than the breakeven frequency of ~0.8 ns^{-1}. Hence, the obtained results should be comparable with those examined in the main body of the study. RMSD of the WPD loop in TMD′ simulation to the open and closed crystal structures was found to be similar to that in TMD_1 simulation (compare Figure S16A with Figure S2C). As expected from a lower spring constant, rotation of type I to type II β-turn did not take place (compare Figure S16B with S13). Two dimensional histogram of Phe182 backbone dihedral angle in TMD′ (Figure S16C) showed a wide distribution along ψ_{182}, similar to that in TMD_1 (compare with Figure S15). Conformational volume occupied by WPD loop in PC subspace (Figure S17A,B) and opening/closing trajectories (Figure S17C) in TMD_1 and TMD′ simulations were found to be highly similar. These findings show that WPD loop motion, except the conformational transition of Asp181-Phe182 backbone, was almost indistinguishable in both TMD simulations at different k values. Overlap of reconstructed in-phase trajectories in both simulations were found to be 0.98 and 0.92 for residues 1 to 280, and for residues on which TMD potential was not directly applied, respectively, indicating that propagation of the local perturbation along the backbone was similar at different k values of TMD simulations. 97% and 93% of C_α atoms (residues 1 to 280) at $\alpha = 0.05$ and 0.01, respectively, in TMD_1 were found to be perturbed also in TMD′ simulation, confirming the robustness of the current method in identifying perturbed atomic variables with respect to different spring constants.
