# Supplementary material for: Frequency Response of a Protein to Local Conformational Perturbations
Source: PLoS Comput Biol. 2013 Sep 26;9(9):e1003238. doi: 10.1371/journal.pcbi.1003238 (PMC3784495; doi:10.1371/journal.pcbi.1003238)
Supplement: Text S6 — Relation between power of a signal and atomic MSF. (PDF) [file pcbi.1003238.s029.pdf]

Text S6. Relation between power of a signal and atomic MSF

In the current study, atomic position, dihedral angle, and atomic distance trajectory signals are processed, so a more convenient measure than energy may be mean square fluctuations (MSF). When the signal is mean-centered, i.e. deviation of the trajectory from its mean value, MSF is equivalent to the power of the signal:

$$\mathrm{MSF} = \frac{1}{N}\sum_{n=0}^{N-1}\left|x_n\right|^2 = \frac{E}{N} = P \tag{S1}$$

$$\mathrm{MSF} = P = \frac{E}{N} = \frac{1}{N^2}\sum_{k=0}^{N-1}\left|X_k\right|^2 = \sum_{k=0}^{N-1}\left|\frac{X_k}{N}\right|^2 \tag{S2}$$

By this way, contribution of kth sinuoidal component to the MSF of the trajectory is found to be equal to $\left|\frac{X_k}{N}\right|^2$. DFT may be applied to each of the three Cartesian coordinates (r = 1,2,3) of an atom.

$$X_n^r = \sum_{n=0}^{N-1} x_n^r e^{-\frac{i2\pi kn}{N}} \tag{S3}$$

In this case, MSF of a single atom, using Parseval's relation, can be obtained by

$$MSF = \sum_{r=1}^{3}\frac{1}{N}\sum_{n=0}^{N-1}\left|x_n^r\right|^2 = \sum_{r=1}^{3}\sum_{k=0}^{N-1}\left|\frac{X_k^r}{N}\right|^2 = \sum_{k=0}^{N-1}\sum_{r=1}^{3}\left|\frac{X_k^r}{N}\right|^2 \tag{S4}$$

Here, the contribution of the kth sinusoidal component to the MSF of an atom (power at the kth frequency component) is equal to $\sum_{r=1}^{3}\left|\frac{X_k^r}{N}\right|^2$. Generalization of this equation to R number of C_α atoms corresponding to a specific region in the protein, contribution of the kth sinusoidal component to the residue-averaged MSF can be computed by $\frac{1}{R}\sum_{r=1}^{3R}\left|\frac{X_k^r}{N}\right|^2$.
