# Supplementary material for: Frequency Response of a Protein to Local Conformational Perturbations
Source: PLoS Comput Biol. 2013 Sep 26;9(9):e1003238. doi: 10.1371/journal.pcbi.1003238 (PMC3784495; doi:10.1371/journal.pcbi.1003238)
Supplement: Text S7 — Time and frequency responses of first order lag and lead-lag systems. (PDF) [file pcbi.1003238.s030.pdf]

Text S7. Time and frequency responses of first order lag and lead-lag systems

Step and frequency responses of a first order lag and a lead-lag system are analyzed to have a better insight of the dynamics of these two systems. Step response of a first order system (with K_p=1) shows an increase from the zero initial position to a constant steady state value, and speed of convergence is determined by time constant τ_p (Figure S18A). Frequency response (Figure S18B) shows that output amplitude converges to a constant value for frequencies lower than the breakpoint frequency (=1/τ_p), while higher frequency inputs are attenuated.

When the lead element in the transfer function is written as a superposition of a gain and a first order lag process (Equation 14), it is easier to determine the contribution of each element to the lead-lag transfer function. The gain element (τ_z/τ_p) will give an instantaneous response, while the contribution of the first order lag element to the output will build up in time, proportional to τ_p. Therefore, if τ_z/τ_p ratio is smaller than unity, then the gain of the first order lag element will be positive and contributions of both elements to the step response will be in the same direction (Figure S18C). If, on the other hand, τ_z/τ_p ratio is larger than unity, then then the gain of the first order lag element will be negative, and the output, again starting from a positive value at t = 0, will move closer to its previous equilibrium state (Figure S18D). For lead-lag systems with τ_z/τ_p ratio smaller than unity, frequency response is similar to that of first-order lag systems for frequencies lower than 1/τ_p (Fig. S18E). For lead-lag systems with τ_z/τ_p ratio higher than unity, input fluctuations are magnified in high frequencies (Figure S18F).
